# A New Class of Pluripotent Stem Cell Cytotoxic Small Molecules

**DOI:** 10.1371/journal.pone.0085039

**Published:** 2014-03-19

**Authors:** Mark Richards, Chee Wee Phoon, Gwendoline Tze Wei Goh, Eng Khuan Seng, Xu Ming Guo, Cherine Mei Fong Tan, Woon-Khiong Chan, Joel Mun Kin Lee

**Affiliations:** 1 School of Chemical & Life Sciences, Nanyang Polytechnic, Singapore; 2 Singapore Eye Research Institute, 11 Third Hospital Avenue, Singapore; 3 Department of Biological Sciences, National University of Singapore, Singapore; 4 Food & Human Nutrition Programme (Singapore), School of Agriculture, Food and Rural Development, Agriculture Building, Newcastle University, Newcastle upon Tyne, United Kingdom; University of Queensland, Australia

## Abstract

A major concern in Pluripotent Stem Cell (PSC)-derived cell replacement therapy is the risk of teratoma formation from contaminating undifferentiated cells. Removal of undifferentiated cells from differentiated cultures is an essential step before PSC-based cell therapies can be safely deployed in a clinical setting. We report a group of novel small molecules that are cytotoxic to PSCs. Our data indicates that these molecules are specific and potent in their activity allowing rapid eradication of undifferentiated cells. Experiments utilizing mixed PSC and primary human neuronal and cardiomyocyte cultures demonstrate that up to a 6-fold enrichment for specialized cells can be obtained without adversely affecting cell viability and function. Several structural variants were synthesized to identify key functional groups and to improve specificity and efficacy. Comparative microarray analysis and ensuing RNA knockdown studies revealed involvement of the PERK/ATF4/DDIT3 ER stress pathway. Surprisingly, cell death following ER stress induction was associated with a concomitant decrease in endogenous ROS levels in PSCs. Undifferentiated cells treated with these molecules preceding transplantation fail to form teratomas in SCID mice. Furthermore, these molecules remain non-toxic and non-teratogenic to zebrafish embryos suggesting that they may be safely used *in vivo*.

## Introduction

The discovery of induced Pluripotent Stem Cells (iPSCs) by Yamanaka and co-workers [1] and improvements in “xeno-free” and feeder-free PSC culture systems [2] have significantly advanced the prospects of PSC-based regenerative medicine therapies. However, 3 major hurdles in regenerative medicine still remain. First, the genetic instability of PSCs needs to be resolved [3]–[6]. Second, scale-up protocols with the capacity to culture PSCs to generate sufficient numbers of cells for clinical application have to be formulated [5], [7]. Third, methods to consistently and completely eliminate teratoma-forming undifferentiated PSCs that potentially contaminate clinically important specialized derivatives need to be derived [5]–[6], [8]–[12].

The third problem of tumor formation by rogue PSCs is particularly worrying as just 100 hESCs are capable of forming teratomas [11]. This estimate has made the complete removal of undifferentiated PSCs for certain disease targets where billions of specialized cells are necessary for transplantation (eg. diabetes and heart conditions) particularly daunting. Indeed the focus for PSC-based cell therapies has shifted somewhat to clinical trials where the transplantation of smaller numbers of cells would be sufficient for a cure, like in the case of eye disease such as Macular Dystrophy where only tens of thousands of PSC-derived Retinal Pigment Epithelial cells are required to restore sight in patients [13].

A variety of strategies have been suggested to eliminate rogue undifferentiated PSCs residing in a pool of differentiated cells. These include the selective removal of undifferentiated PSCs in the heterogeneous cell population via flow cytometry [14], the development of cytotoxic antibodies that specifically target undifferentiated PSCs [15], [16], the separation of undifferentiated PSCs from the differentiated cells prior to transplantation using cell sorting methods alone or in combination with density gradient separation [17], [18], deliberate extended differentiation of PSC-derivatives to allow residual undifferentiated PSCs to differentiate into an undesirable cell type [19], and the creation of transgenic PSC lines with suicide genes that can be activated at will to selectively remove undifferentiated cells before transplantation [9], [20].

Many of these solutions are antibody-based. However, while antibodies have high specificity they are relatively difficult and costly to produce. Their routine use in enrichment protocols to process large numbers of cells will also escalate the cost of clinical application. Processing of cells for FACS or MACS is also lengthy and time consuming and may ultimately lead to cell attrition and loss of cell viability.

## Results

**Figure 1:**
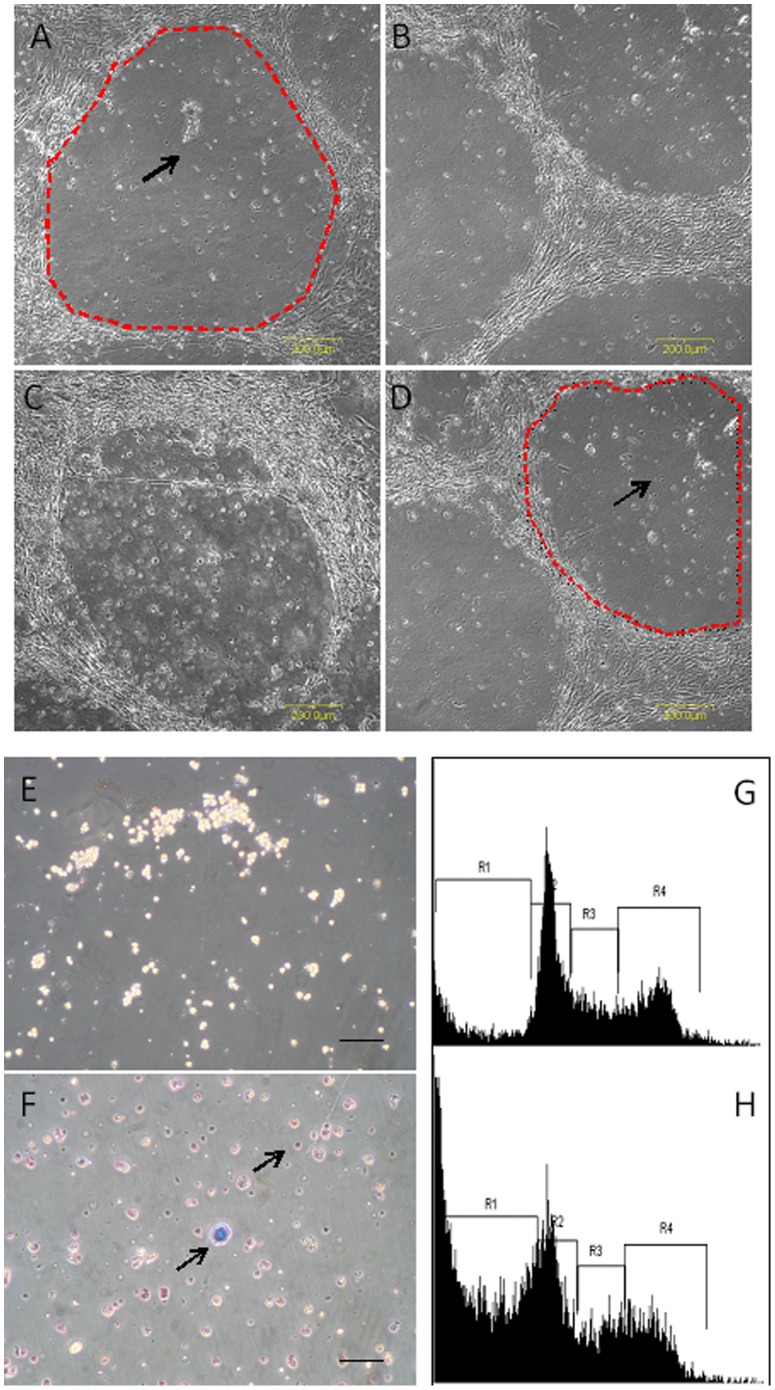
Morphology of BGO1V-MEF cultures following treatment with 20 mM JC011 for 12 hrs. “Hollowing-out” effect in BGO1V-MEF cultures, feeders remain intact and viable (**A–D**). Trypan blue staining of untreated control BGO1V single cells (**E**). Trypan blue staining of JC011 treated BGO1V single cells showing an increase in Trypan blue uptake (**F**). Propidium Iodide DNA content analysis of control untreated BGO1V cells (**G**). Propidium Iodide DNA content analysis of JC011 treated BGO1V after 6 hrs showing a rapid increase in the sub-G1 fraction (**H**). R1 = sub-G1 fraction, R2 = G1 fraction, R3 = early/late S-phase fraction, R4 = G2/M fraction.

To overcome the issues associated with existing antibody-based enrichment paradigms, we conducted a large-scale screen with the BGO1V hESC line supported on Mouse Embryonic Fibroblast (MEF) feeders to identify PSC-specific cytotoxic small molecules. The PSC-MEF co-culture system comprises 2 morphologically distinct cell types, spindle-shaped MEF feeders which are a mixed population of differentiated cells and colony forming hESCs with a high nucleus to cytoplasm ratio representing healthy undifferentiated PSCs. These differences in morphology as well as the unique arrangement of these 2 distinct cell types (MEFs surrounding distinct hESC colonies) in the co-culture monolayer helps facilitate the visual identification of compounds which may be specifically cytotoxic to hESCs. A “hollowing-out” effect of the co-culture would be indicative of differential cytotoxicity (Fig. 1a–d).

Briefly, all BGO1V-MEF co-cultures were treated with 10 µM final concentration of compounds for 12 hours after which visual inspection of each well was performed under a stereomicroscope to identify small molecules that were cytotoxic to BGO1V but which would leave the MEFs intact.

**Figure 2:**
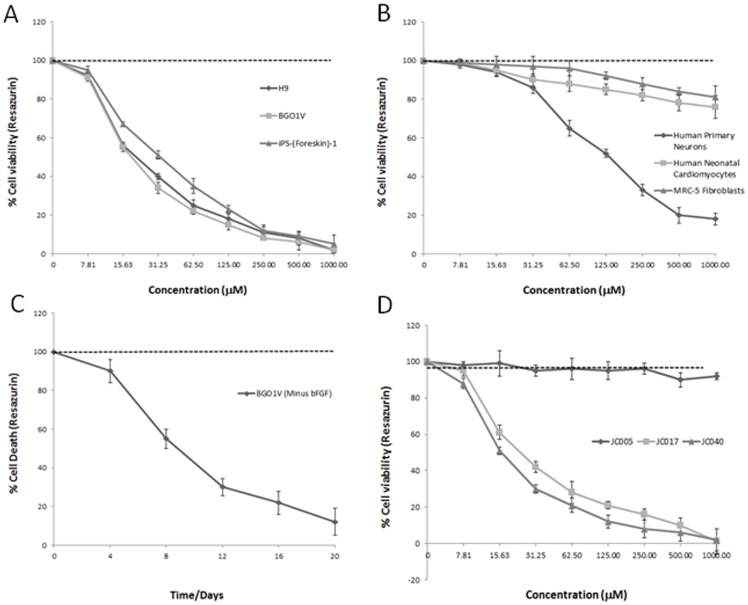
Dose response cytotoxicity data. Dose response curves for 3 PSC cell lines (BGO1V, H9 and iPS-Foreskin-1) following treatment with JC011 (**A**). Dose response curves for 3 specialized somatic cell lines (MRC-5, human primary neurons and human neonatal cardiomyocytes) treated with JC011 (**B**). Time course cytotoxicity analysis for differentiating BGO1V cultures treated with JC011 at 4-day intervals following bFGF withdrawal (**C**). Dose response curves for 3 JC011 analogues (JC005, JC017 and JC040) showing that JC040 with a longer alkyl side-chain is the most potent analogue (**D**). Cell viability values were normalized to untreated controls and reported as mean ± S.D. of three independent experiments (n = 3).

From the screens, 3 structurally related compounds (JC010, JC011, JC017) from an in-house synthesized compound library that displayed differential cytotoxicity towards PSCs were identified (Fig. 2a). Cell viability analysis was performed using the Resazurin dye, confirmed via Trypan Blue staining (Fig. 1e, f) and Propidium Iodide DNA content analysis (Fig. 1j, k). Dose response relationships for each of these 3 active hits were generated using feeder-free cultures for 3 PSC lines (BGO1V, H9 and iPS-foreskin-1) (Fig. 2b). PSC-specific cytotoxic effects for these 3 compounds were found to be consistent across all 3 PSC lines we evaluated. Overall, JC011 was found to be the most potent, 12 hr treatment with 10 µM of JC011 resulted in over 99% cell death of BGO1V cells in feeder-free culture (Fig. 2a). Conversely, cell viability figures for MRC-5 normal human fibroblasts treated with JC011 at concentrations of up to 100 µM remained very high at over 97% in all cases (Fig. 2b). Average IC_50_ value for the 3 PSC lines tested was approximately 20 µM (Fig. 2a). JC011 was also cytotoxic to the NCCIT embryonic carcinoma line with an IC_50_ value of approximately 30 µM.

A time-course experiment to assess how quickly JC011 would induce cell death in the 3 PSC lines used in this study indicated that maximum cell death of about ∼96% would be attainable following a 36 hr incubation with 20 µM JC011 (Figure S5 in File S1).

To confirm its specificity for PSCs, we obtained dose response relationships for JC011 across 4 normal human primary specialized cells types ie. MRC-5 fibroblasts, primary human astrocytes (Lonza), primary human neurons (Neuromics) and primary human neonatal cardiomyocytes (National Heart Centre, Singapore). Low cytotoxicity (<5% cell death) towards MRC-5, primary cardiomyocytes and primary astrocytes were observed for JC011 concentrations of up to 100 µM (Figure S2 in File S1). Primary human neurons were found to be substantially more sensitive to JC011 treatment with cell vitality affected at concentrations of 60 µM (Fig. 2b). We further tested JC011 at the IC_50_ concentration of 20 µM on several other human cell lines (HepG2, HeLa, WI-38 and normal human keratinocytes) and found that resultant cell viability as determined by Resazurin analysis remained high at >97.5% (Figure S6 in File S1). Additionally, all PSC lines used in this report were >92% positive for both Tra-1-60 and SSEA-4 as established by FACS thus confirming specificity of JC011 towards PSCs (Figure S7 in File S1).

JC011 was also tested on differentiating BGO1V/hOG cultures harbouring the hOct4::GFP construct. BGO1V-GFP cultures were induced to differentiate by bFGF starvation; differentiating cultures were treated at 4-day intervals with 20 µM of JC011 and cell viability determined via Resazurin analysis. A clear trend was observable in differentiating BGO1V/hOG cultures that were treated with JC011. Decreasing GFP levels indicative of increasing PSC differentiation were contemporaneous with an overall increase in cell viability, thus confirming specificity of JC011 for the undifferentiated PSC phenotype (Fig. 2c).

**Figure 3:**
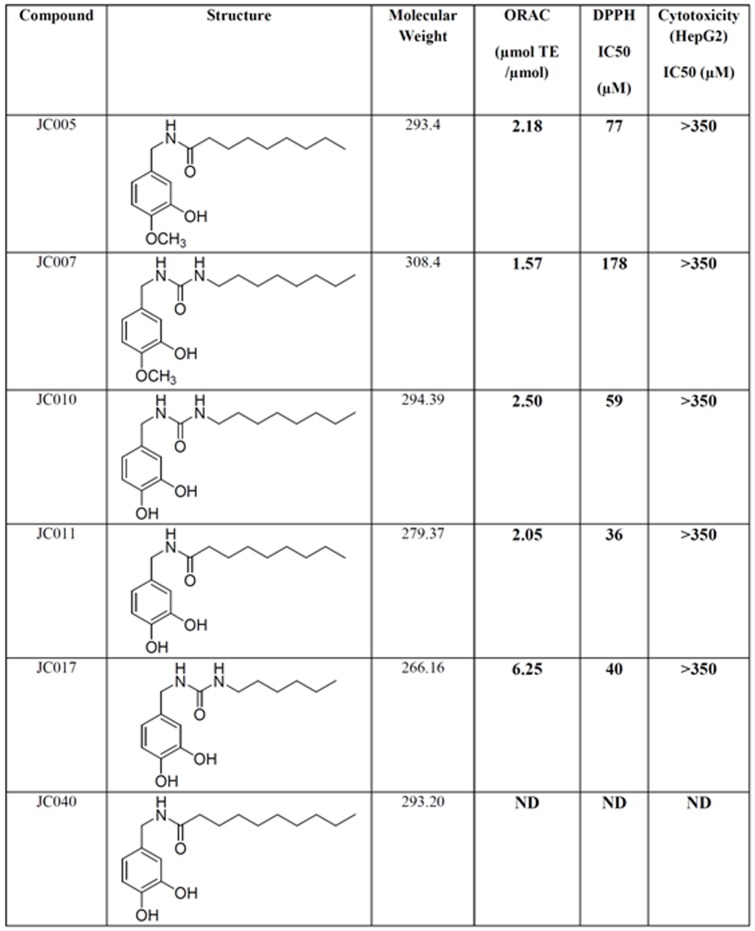
Table of structure, molecular mass, ORAC and DPPH antioxidant data for 6 JC analogues (JC005, JC007, JC010, JC011, JC017 and JC040).

Structure-activity relationship studies on the active hits were carried out to determine the functional groups that are responsible for eliciting the activity and also to optimise the potency of these molecules. A focused library of analogues with different substituents at the aromatic ring, linker and varying lengths of alkyl side-chains were screened (Fig. 3). Our studies show that it is crucial to have 2 hydroxyl groups present at the carbon-3 and -4 positions on the aromatic ring. As shown by the data of JC005 and JC007, replacement of any of these hydroxyl groups with a methoxy (CH_3_O-) group led to a significant drop of cytotoxicity towards PSCs and specialised cells (Fig. 2d).

The activity can also be affected by varying the length of the alkyl side chains. JC040, with a 9-carbon side chain, has an IC_50_ value of approximately 16 µM (Fig. 2d) and it is slightly more potent than JC011. However, extending the alkyl side chain to 10 carbons (JC049) and longer (JC048, JC050) generally decreased the potency. This could be attributed to an increase in lipophilicity which decreases their aqueous solubility. Even though JC040 showed a higher potency, JC011 was selected as our model compound for further screens as it has a better solubility profile. The nature of the linker between the alkyl side chain and the aromatic core was also investigated. It was shown that JC011 which has an amide linkage is more potent, as compared to JC010 which has a urea (-NHCONH-) linkage.

**Figure 4:**
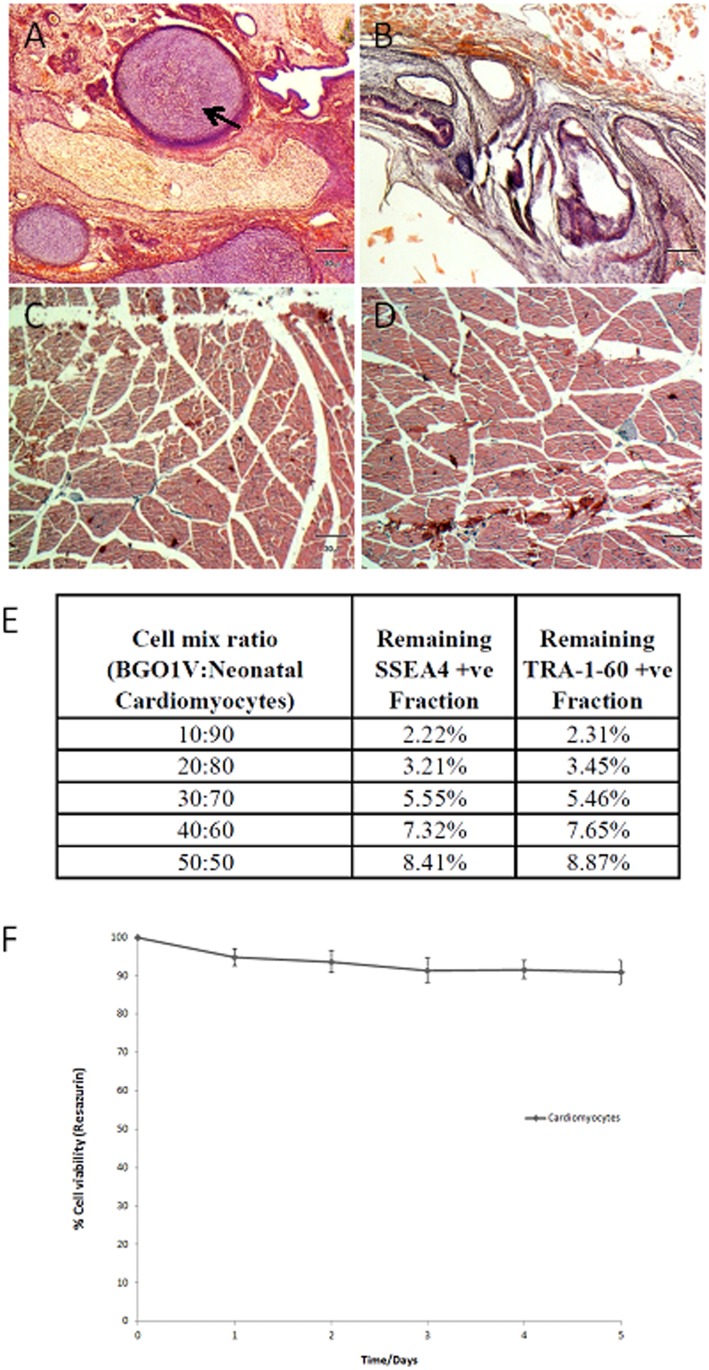
JC011 treated PSCs fail to form teratomas in SCID mice and can be effectively used to enrich for specialized cells. Teratoma sections from SCID mice. Controls showing typical teratoma tissue organization representative of all 3 germ layers, A = Cartilage, B = Gut/Intestine, C, D = JC11 treated sample showing plain muscle tissue with no teratoma growth (**A–D**). BGO1V and primary neonatal cardiomyocytes were mixed and seeded in pre-determined ratios (10∶90, 20∶80, 30∶70, 60∶40 and 50∶50). The mixed cultures were treated with JC011 at 20 µM for 12 hrs followed by SSEA-4 and TRA-1-60 FACS analysis to determine enrichment ratios (**E**). Time course cell viability data for JC011 (20 µM) treated primary neonatal cardiomyocytes indicate that cardiomyocytes maintain high cell viability (>95%) even after a 5-day incubation period with JC011 (**F**).

To establish if treatment of hESCs with JC011 would abolish teratoma formation *in vivo*, we treated 1×10^6^ BGO1V cells with 20 µM JC011 for 12 hrs prior to injecting the treated cells into the thigh muscle of SCID mice. JC011-treated hESCs failed to form teratomas in all SCID mice while untreated controls readily formed teratomas (Fig. 4). To better simulate the efficacy of a JC011-based enrichment strategy as part of a differentiation protocol, heterogeneous populations of BGO1V and primary neonatal cardiomyocytes or astrocytes were mixed in pre-determined ratios (10∶90, 20∶80, 30∶70, 60∶40 and 50∶50). The mixed cultures were seeded, allowed to adhere for 14 hrs and subsequently treated with JC011 at 20 µM for both BGO1V-cardiomyocyte cultures and BGO1V-astrocyte cultures for 12 hrs after which cultures were analysed by FACS for SSEA-4 and TRA-1-60 expression to determine enrichment percentages. Up to 6-fold enrichment for differentiated cardiomyocytes and astrocytes were obtained from the different mixed cell populations following JC011 treatment without adversely affecting cell vitality (Fig. 4e). These results are an improvement over the 4.5-fold enrichment figures obtained by single antibody sorting using the classical hESC SSEA-4 or TRA-1-60 antibodies [14]. No further increase in cardiomyocyte cell death beyond 5% was observed following prolonged exposure (up to 5 days) to 20 µM JC011 (Fig. 4f).

To better understand the toxicity of JC011 *in vivo* and in a whole animal model, acute toxicity (LC_50_) for JC011 was determined in zebrafish. The results suggest that JC011 was toxic to zebrafish embryos only at very high concentrations (JC011 LC_50_ = 398.9 µM) (Figure S3 in File S1). JC011 LC_50_ values for zebrafish embryos are comparable in magnitude to the reported values for several FDA approved drugs such as Gentamycin Sulfate (440 µM) and Verapamil Hydrochloride (170 µM) [21].

In order to further assess developmental toxicity of JC011, its maximum non-lethal concentration (MNLC) was determined by exposing developing zebrafish to JC011 from the early gastrula stage at 6 hours post fertilization(hpf)to 5 days post fertilization (dpf). MNLC for JC011 was determined at approximately 425 µM. Zebrafish were treated at MNLC from 6 hpf to 5 dpf and visually assessed using a stereomicroscope. At 425 µM (MNLC), 21.1% (4/19) malformations were observed. Zebrafish treated with JC011 exhibited accidental incidences of trunk/tail/notochord, liver and intestine malformation, but these figures were not statistically significant (p>0.05) (Figure S3 in File S1). These data confirm that JC011 is not developmentally toxic to developing zebrafish embryos from the gastrula stage onwards and support the finding that JC011 toxicity is confined to very early embryonic cells.

**Figure 5:**
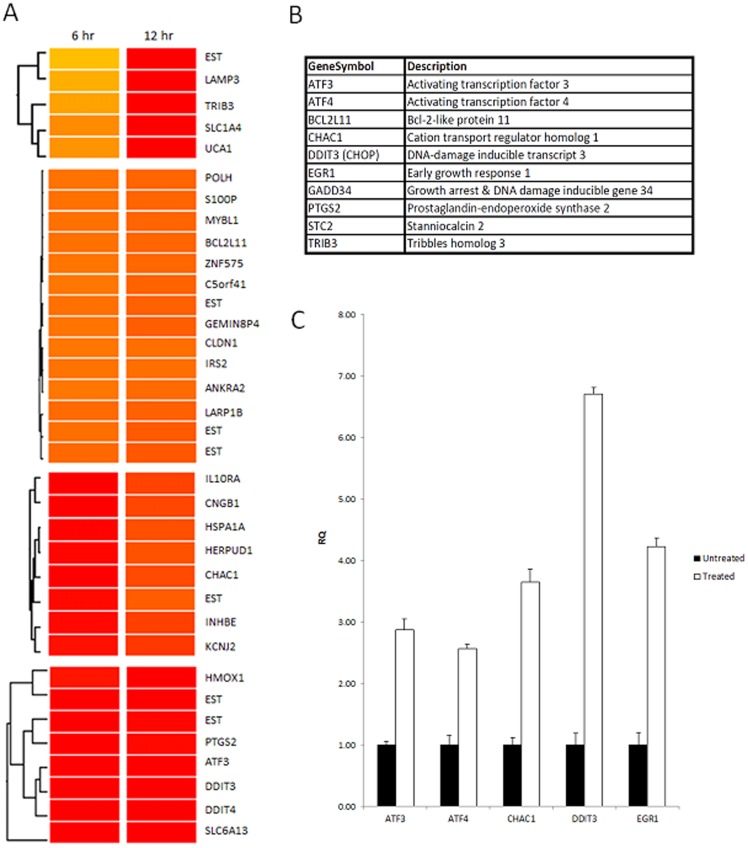
Comparative microarray analysis reveals involvement of the PERK/ATF4/DDIT3 ER stress pathways. Clustering of key differentially upregulated ER stress genes in BGO1V following 6 µM JC011 (**A**). Top 10 components of the PERK/ATF4/DDIT3 ER stress pathway that were found to be rapidly upregulated in JC011 treated BGO1V cells (**B**). qRT-PCR confirmation of upregulated UPR/ER stress pathway genes following JC011 treatment (**C**).

Comparative gene expression profile analysis with microarray was next performed to elucidate the mechanisms of JC011-mediated PSC cytotoxicity. Total RNA from JC011-treated BGO1V cultures was extracted at 6 hr and 12 hr time-points and used for gene expression analysis while total RNA from untreated BGO1V cultures served as controls. We found rapid upregulation of genes associated with the unfolded protein response (UPR) also known as the endoplasmic reticulum stress response (ER stress) in 6 hr and 12 hr JC011-treated cultures. More than 10 ER stress related genes were found to be present in the top 50 upregulated list of genes (Fig. 5).

The ER stress response, also known as the unfolded protein response (UPR), is a cellular stress mechanism activated in response to an accumulation of mis-folded proteins in the lumen of the endoplasmic reticulum [22], [23]. In conditions of prolonged stress, UPR commits the cell to a pathway of apoptosis. Signalling intermediates downstream of all 3 main UPR receptor pathways have been identified as having pro-apoptotic roles [22], [23]. The 3 principal UPR receptors involved in the UPR apoptosis cascade are Ire1, ATF6 and PERK. Microarray analysis further revealed that the downstream components and genes of the PERK/ATF4/DDIT3 pathway in particular were specifically upregulated in JC011-treated BGO1V cells. DDIT3 (also known as CHOP), CHAC1, ATF3 and ATF4 are key mediators of PERK mediated UPR apoptosis; all 4 of these genes were found to be very highly upregulated and this was confirmed by qRT-PCR (Fig. 5).

**Figure 6:**
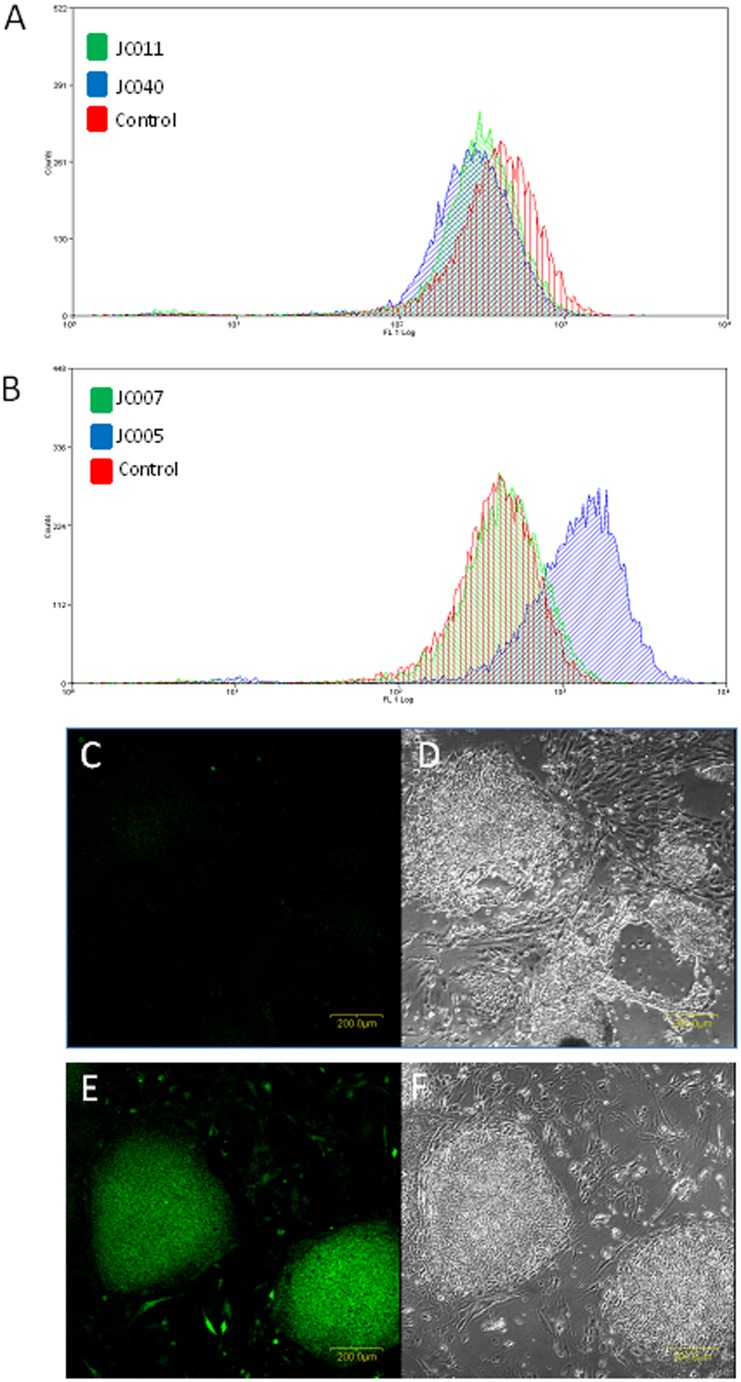
JC011 and JC040 reduce ROS levels in PSCs. Cytotoxic JC011 and JC040 induce a small but rapid reduction of intracellular ROS levels in BGO1V cells as confirmed by DCHF-DA FACS analysis 3 hrs after treatment (**A**). JC007 (non-cytotoxic) does not alter endogenous ROS levels while JC005 (non-cytotoxic) increases ROS levels but without any corresponding cytotoxicity to BGO1V (**B**). FACS histograms are representative outcomes of 4 independent experiments. DCHF-DA stained JC011 treated BGO1V cells (**C, D**). DCHF-DA stained JC005 treated BGO1V cells (**E, F**).

Moreover, microarray data also indicated fluctuations in oxidative stress and calcium signalling in JC011-treated BGO1V cells. GCLM and GSR, 2 genes involved in Glutathione metabolism were found to be rapidly upregulated following JC011 treatment. Changes in intracellular ROS levels following JC011 treatment were examined with the fluorescent ROS sensitive dye DCHF-DA. Unexpectedly, ROS levels in DCHF-DA cells were found to be rapidly reduced following JC011 treatment in BGO1V cells after a short incubation period of 3 hrs. In contrast, ROS levels in BGO1V cells were not reduced following treatment with the non-cytotoxic JC005 and JC007 analogues although both JC011 and JC005 share similar antioxidant profiles as determined by the Oxygen Radical Absorbance Capacity (ORAC) and Di(phenyl)-(2,4,6-trinitrophenyl)iminoazanium (DPPH) antioxidant assays (Fig. 6 & Fig. 3). Instead, ROS levels were increased following JC005 treatment but this increase was not associated with any observable cell death.

Next, pharmacological intervention with a well-studied ER stress inducer dithiothreitol (DTT) was performed to determine if JC011 mediated PSC cytotoxicity was a specific outcome of JC011 treatment or a more general non-specific outcome which could be replicated by any UPR/ER stress inducer. Treatment with DTT, which causes disulphide bond disruption in ER proteins, did not result in any observable increase in BGO1V cell death when compared to untreated controls. PSC-specific cytotoxicity in BGO1V cells could not be replicated with DTT treatments confirming that JC molecule mediated PSC-specific cytotoxicity is a property specific to the JC molecule series (Figure S8 in File S1).

**Figure 7:**
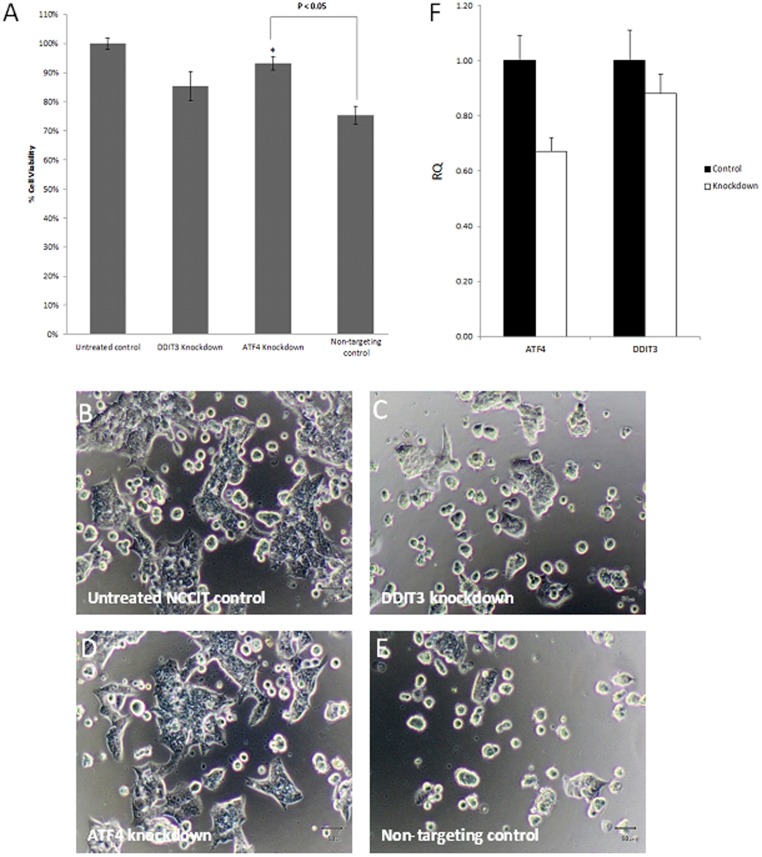
siRNA knockdown of the key ER stress genes ATF-4 and DDIT3 leads to reduced sensitivity towards JC011 in NCCIT cells. ATF-4 and DDIT3 were silenced via siRNA knockdown. Sensitivity towards JC011 was attenuated in DDIT3 knockdown and ATF-4 knockdown (P<0.05) NCCIT cells thereby confirming involvement of the PERK/ATF4/DDIT3 ER stress pathway in JC011 mediated cytotoxicity (**A–E**). qRT-PCR confirmation of ATF-4 and DDIT3 transcript knockdown (**F**). Cell viability figures were normalized to untreated controls and reported as mean ± S.D. of three independent experiments (n = 3). Statistical analysis was performed with the Student's T-test.

We then performed siRNA knockdown on 2 genes, DDIT3 and ATF4 to confirm the involvement of PERK/ATF4/DDIT3 ER stress pathway in JC011 mediated PSC-specific cytotoxicity. siRNA knockdown was carried out in NCCIT cells for maximum transfection efficiency and confirmation of transcript knockdown was determined by qRT-PCR. Briefly, NCCIT cells were allowed to recover for a period of 24 hrs following siRNA knockdown and treated with JC011. Cell viability analysis indicated that ATF4 knockdown successfully attenuated JC011 cytotoxicity in NCCIT cells (P<0.05) thereby confirming the role of PERK/ATF4/DDIT3 ER stress in JC011 induced PSC cytotoxicity. DDIT3 knockdown NCCIT cells also showed a similar attenuated cytotoxic response when challenged with JC011 but these data were not statistically significant (Fig. 7). This result could perhaps be due to other components of PERK/ATF4/DDIT3 ER stress pathway which may potentially substitute for loss of DDIT3 activity. ROS levels determined by DCHF-DA FACS were used as surrogate readout to confirm knockdown of ATF4 and DDIT3. ATF4 knockdown resulted in a recovery of ROS levels comparable to untreated controls while DDIT3 knockdown resulted in no significant ROS recovery (Figure S9 in File S1)

## Discussion

Considerable progress has been achieved in establishing optimum conditions to propagate and differentiate PSCs into a variety of lineages of functional specialized cells for human cell replacement therapies. These advancements are further underscored by recent U. S Food and Drug Administration approval of clinic trials to use PSC-derived cells to treat spinal cord injury and macular degeneration [24], [25]. Nonetheless, the teratoma risk associated with contaminating PSCs in differentiated cell populations still remains. This problem is further aggravated by the intrinsic propensity for some PSC derivatives to undergo dedifferentiation *in situ* [4], [11]. Even if ensuing data from clinical trials supports the efficacy and safety of the PSC-based therapies, the teratoma risk might have to be continuously monitored when PSC-based therapies are routinely applied in a clinical setting.

Antibody-based solutions to remove undifferentiated PSCs *in vitro* are currently popular. The high specificity of an antibody towards a particular antigen also has its drawbacks as any antibody-based strategy could in theory be limited by the specificity of the expression of its associated surface antigen. It is well documented that PSC cultures comprise of a mixed population of cells with slightly different phenotypes [3]. Consequently, the undifferentiated cells from one sample may not express similar groups of surface antigens; this would necessitate the use of multiple combinations of antibodies for any antibody-based enrichment strategy. In addition, Fluorescence Activated Cell Sorting (FACS) or Magnetic Activated Cell Sorting (MACS) based enrichment protocols require single-cell suspensions. Manipulation of single cells may not be appropriate in all tissue engineering scenarios especially when complex 2-D or 3-D multi-layer cell constructs are the final product for transplantation.

It is also difficult to envisage the use of an antibody as a preventive drug in an established clinical transplantation regime where patients may require administration of a drug for a period of time following the transplantation procedure to prevent teratoma formation. Small molecules which are specifically cytotoxic to PSCs and non-toxic to normal specialized cells could be a better solution in these scenarios. Small molecules are cheaper and easier to synthesize. They also show favourable pharmacokinetic profiles and elaborate FACS or MACS set-ups and protocols are also not required.

While this manuscript was in preparation, a high throughput screen study of over 50,000 small molecules reported the identification of 15 pluripotent cell-specific inhibitors (PluriSIns) [26]. Interestingly, Ben-David *et al* determined that their most selective PluriSIn induces ER stress apoptosis in PSCs [26]; we corroborate these findings in identifying the ER stress pathway as a new target for understanding the signalling cascades which govern apoptosis in PSCs.

However, in contrast to the PluriSIns described in the study by Ben-David *et al*, our compounds (JC011, JC010, JC017 and JC040) may not be involved in lipid metabolism as our molecules are structurally different and do not share a phenyl-hydrazine moiety which is a common feature of 9 PluriSIns [26]. Instead, the 4 JC compounds we describe have high ORAC and DPPH values indicative of strong anti-oxidant properties (Fig. 3). We also establish that JC molecule PSC cytotoxicity is mediated mainly through the PERK/ATF4/DDIT3 signalling arm of the ER stress pathway and concomitant with a small but rapid reduction of intracellular ROS levels. This latter observation is unexpected because cell death as a result of the ER stress response is more commonly associated with an increase rather than a decrease in endogenous intracellular ROS levels [22], [23].

We present in this report a group of novel small organic molecules that are cytotoxic and effective against PSCs. These molecules can be easily and efficiently synthesized besides having several advantages over existing antibody-based enrichment strategies for the removal of PSCs from their differentiated progenies. These molecules should also be useful as an *in vitro* tool to help enrich certain differentiated PSC derivatives by eradicating contaminating undifferentiated PSCs. Although toxicity towards primary cardiomyocytes (∼5–9%) was observed following treatment with JC011 (20–100 µM) and toxicity towards astrocytes (<5%) observed at 20 µM, this should be acceptable as JC011 concentrations of <20 µM were found to be sufficient in reducing PSC contamination in differentiated cell populations by up to 6-fold (Fig. 4e). Additionally, prolonged exposure of cardiomyocytes to JC011 (20 µM) for up to 5 days resulted in no further increase in cell death. These statistics are an improvement over the 4.5-fold enrichment figures obtained by single antibody sorting using classical hESC antibodies [14]. Primary neurons however were found to be significantly affected by JC011 at 20 µM; this finding could severely limit the use of the JC compounds in neuronal differentiation strategies.

Furthermore, we corroborate the findings of Ben-David *et al* [26] in identifying the ER stress pathway as a new target for understanding the signalling cascades which govern apoptosis in PSCs. A deeper understanding of the PSC apoptosis machinery will provide an effective means to eliminate teratoma-forming cells from cell preparations with clinically desirable phenotypes, triggering selective apoptosis in PSCs could potentially be one solution to eliminate unwanted teratoma-forming cells. Although additional safety studies have to be performed, our findings indicate that the molecules we describe may perhaps have the potential to be used safely *in vivo*. Downstream, together with other enrichment methods such as antibody-based sorting, these molecules could perhaps function as part of a strategy to safeguard against teratoma formation in PSC-based cell replacement regimes.

## Materials and Methods

### Ethics Statement

This study was carried out in strict accordance with the recommendations of the Responsible Care and Use of Laboratory Animals guide of the National University of Singapore and Nanyang Polytechnic. All animal work was conducted according to relevant Singapore and international NACLAR guidelines at the ASTAR Biological Resource Centre, Singapore. The animal experimentation protocol was approved by the Institutional Animal Care and Use Committee of the National University of Singapore (IACUC Permit No 090446). All surgery was performed under sodium pentobarbital/ketamine mix anesthesia and all efforts were made to minimize suffering. Only commercially available pluripotent stem cell lines were used in this research. Approval for pluripotent stem cell research was obtained from the National University of Singapore Institutional Review Board (NUS-IRB Review Permit No 09-301).

### Cell Culture Protocols

All PSC lines were cultured in Dulbecco's modified Eagle's medium/Ham's F-12 medium containing GlutaMax supplemented with 20% serum replacement, 1 mM sodium pyruvate, 0.1 mM nonessential amino acids, (all from Life Technologies), 0.1 mM β-mercaptoethanol (Sigma), and 8 ng/ml basic fibroblast growth factor (Chemicon). PSCs were grown on primary mouse embryonic fibroblasts (MEFs) obtained from E12.5 d.p.c. 129sv mice. Colonies were serially passaged in “bulk culture” format using Collagenase IV (Life Technologies) and moved to feeder-free culture in mTeSR™ medium (Stem Cell Technologies) when required. PSC lines between passages 30 and 35 were used in this study. HepG2, HeLa, WI-38, Human Keratinocytes, MRC-5 and NCCIT cells were cultured as recommended by American Type Culture Collection (ATCC). Primary hN2™ Human Neurons (Neuromics) were grown in hN2™ human neuron culture media (Neuromics). Primary human neonatal cardiomyocytes (Celprogen) were grown D10 media. Primary human astrocytes (Lonza) were cultured in specialized human astrocyte media (Lonza).

### FACS and Propidium Iodide DNA Content Analysis

Cells were washed with PBS followed by fixation with ice-cold 70% ETOH for 5 min. Fixed cells were washed with PBS twice and resuspended in 300 µl Propidium Iodide solution (69 µM Propidium Iodide, in 38 mM NaCitrate from Sigma). 20 µl of 10 µg/ml RNase was added to remove interfering RNA. Following incubation at 37°C for 45 min, DNA content was determined via FACS analysis using a Beckman Coulter Cell Lab Quanta SC. SSEA-4 and TRA-1-60 antibodies used for FACS were obtained from Chemicon. All experiments were performed in triplicate.

### Trypan Blue Cell Viability Analysis

Cell suspensions were diluted 1∶1 with 10 µl 0.4% w/v Trypan Blue Solution, incubated for 10 min at room temperature and observed under 20× phase optics in a hemocytometer for cell counting.

### Resazurin™ Cell Viability Analysis

Cells were seeded in 96 well multi-well dish format at a density of 10,000 cells per well. Cells were washed with PBS before Resazurin™ (Sigma) solution (40 µg/ml final concentration) was added to each well. Following an incubation period of 2 hrs at 37°C before fluorescence readouts were obtained a Tecan™ plate reader (Emission wavelength: 590 nm, Excitation wavelength: 535 nm, Gain = 25). All experiments were performed in quadruplicate.

### Total RNA Isolation and First-Strand cDNA Synthesis

Total RNA was extracted using TRIZOL (Life Technologies) and reverse transcribed using the SuperScript II™ first-strand synthesis system (Life Technologies). Total RNA was quantified using a ND-1000 spectrophotometer (NanoDrop Technologies, Rockland, DE, USA) and contaminating genomic DNA removed with DNA-free™ reagent (Life Technologies) before reverse transcription.

### qRT-PCR

Real-time quantitative reverse transcriptase polymerase chain reaction (qRT-PCR) analysis was conducted using the ABI PRISM 7500 Fast Sequence Detection System using Predesigned Assays on Demand TaqMan™ probes and primer pairs obtained from Life Technologies. After an initial incubation step for 2 minutes at 50°C and denaturation for 10 minutes at 95°C, qRT-PCR was carried out using 40 cycles of PCR (95°C for 15 seconds, 60°C for 60 seconds). Equal amounts of input first strand cDNA were used for all qRT-PCR reactions, reactions were performed in quadruplicate, and GAPDH RNA levels served as internal controls. Changes in gene expression levels were calculated using the 2dd_CT_ method. Student's T-test statistic was used to analyze qRT-PCR data.

### Microarray

hESCS used for the microarray experiment were grown feeder-free and were >95% positive for the Tra-1-60 marker. Cell viability analysis with Resazurin prior to treatment with JC011 showed all cells to be >98.5% viable. RNA was extracted using standard Trizol™ protocol and subjected to spectrophotometric measurement (BioSPEC-Mini, Shimadzu) and quality determined using an Agilent Bioanalyzer. All samples show OD260/OD280 ratios of between 1.78–2.02 with concentrations ranging from ∼600 ng/µL to ∼980 ng/µL. Results from an Agilent Bioanalyzer analysis showed that all the samples had the RIN (RNA integrity Number) of 7.5 to 9.5. The Agilent™ two-colour microarrays were used for comparative microarray analysis. cRNA was prepared according to the manufacturer's recommended protocol (Agilent™ Two-Color Microarray-Based Gene Expression Analysis, Quick Amp Labeling v5.7). Normalization and data analysis was done using GeneSpring GX version 10.0 and Microsoft Excel. All experiments were performed in triplicate (N = 3). A gene was considered differentially expressed if fold-difference relative to controls was ≥3.

### DCHF-DA ROS Detection, ER Stress Induction and N-acetyl cysteine treatment

DCHF-DA solution was used at a final working concentration of 10 µM. Adherent cells in PBS solution were incubated 5 min room temp with DCHF-DA after which cells were visualized under fluorescence microscope with FITC filters. Bright green fluorescing cells were indicative of cells with high ROS content. To induce non-specific ER stress, DTT (Sigma) was added at a final concentration of 3 mM. All experiments were performed in triplicate. All DCHF-DA experiments were performed after a 3 hr incubation period with JC011. Cells were treated with ROS scavenger N-acetyl cysteine (Sigma) at a final concentration of 100 µM.

### Teratoma Formation in SCID mice

Approximately 1 million stem cells were injected intramuscularly into the right hind leg of a 10-week-old male SCID-beige mouse. 5 mice were used for each experimental arm including controls. Mice were monitored 2–3 times/week for a period of 15 weeks and the size of the teratomas recorded weekly. Histological evaluation of paraffin embedded teratoma sections was performed with H&E staining by a trained pathologist. All animal experiments were carried out in strict accordance with the recommendations of the Responsible Care and Use of Laboratory Animals guide of the National University of Singapore and Nanyang Polytechnic.

### Zebrafish Acute Toxicity Determination

Dechorionated wild-type AB strain zebrafish were distributed into 12-well plates in 3 ml fresh fish water. To determine LC_50_, zebrafish were treated from 2 dpf to 5 dpf. Ten concentrations: 0.01, 0.05, 0.1, 0.5, 1, 5, 10, 50, 100 and 500 µM were assessed to estimate LC_50_. Dead zebrafish were counted and removed daily. At the end of treatment, total number of dead animals was used to generate a lethality curve by plotting % lethality vs concentration. Based on lethality curves, LC_50_s were estimated through logistic regression with the JMP8 software. Experiments were performed 3 times to obtain mean and SD of LC_50_. Detailed protocols are provided in the File S1. All zebrafish acute toxicity assays were performed by a contract service provider, GenScript Pte Ltd.

### Zebrafish Teratogenicity Determination

Chorionated wild-type AB strain zebrafish were distributed into 6-well plates in 3 ml fresh fish water. To determine developmental toxicity, zebrafish were treated at MNLC from 6 hpf (Gastrula stage) to 5 dpf. Zebrafish treated with 0.1 µM 9-cis retinoic acid served as positive control and 0.1% DMSO as vehicle control. Untreated zebrafish were used to demonstrate vehicle (0.1% DMSO) did not have adverse effect on zebrafish. At the end of treatment, zebrafish were visually assessed using a dissecting microscope for malformations. A zebrafish exhibiting defects in any end point was considered abnormal and % abnormal animal for each compound was determined using the following formula: % abnormal zebrafish = (number of abnormal zebrafish/20)×100%. The number of zebrafish exhibiting at MNLC was counted and compared with vehicle control. Fisher's exact test was used to analyze the data; a compound was considered teratogenic when significant difference (p<0.05) was observed in compound treated zebrafish. Detailed protocols are provided in supplementary material. All zebrafish developmental toxicity assays were performed by a contract service provider, GenScript Pte Ltd.

### siRNA Transfection

Cells were seeded in 24-well plates 48 hours before siRNA transfection in order to have 60–70% confluency on the day of transfection. Cells were transfected with siRNA (Dharmacon) targeting (ATF4 and DDIT3) and control siRNA at a final concentration of 100 nM using DharmaFECT 2 reagent (Dharmacon). ON-TARGET plus siRNA pool (Dharmacon) was used for all knockdown experiments because it contains a pool of four individual siRNA sequences for the target gene. RNA knockdown was confirmed by assessing mRNA levels with qRT-PCR.

### Preparative HPLC Protocol

Preparative HPLC was performed on a Shimadzu LC-8A HPLC system equipped with a CBM-20A PDA detector, Gilson 215 liquid handler and fraction collector using a X-Bridge Prep C_18_ (30 mm I.D.×50 mm, 5 µ) column. An isocratic elution with 20% CH_3_CN, 80% (0.1% formic acid/H_2_O) at a flow rate of 20.0 mL/min for 5 min was used. This was followed by a gradient elution starting with 20% CH_3_CN, 80% (0.1% formic acid/H_2_O), and ending with 100% CH_3_CN at a flow

### Liquid Chromatography-Mass Spectrometry (LC-MS)

LC-MS data of JC005, JC007, JC010, JC011, JC017 and JC040 were collected on a Shimadzu LCMS-IT-TOF instrument equipped with SPD-M20A PDA detector, LCMS-IT-TOF MS detector and LC-20AD binary gradient pump using a Shimpack VP-ODS (2.0 mm I.D.×150 mm) column (File S1). An isocratic elution with 20% H_2_O and 80% CH_3_CN at a flow rate of 0.2 mL/min over 3 min was used.

### Nuclear Magnetic Resonance (NMR) Spectroscopy

^1^H and ^13^C NMR spectra of JC010, JC011 and JC040 were recorded on a Bruker AVANCE-400 NMR spectrometer (File S1). The compounds were dissolved in CD_3_OD.

### Synthetic Procedure for Analogues JC005, JC011, JC040, JC048-050

To a solution of the hydrochloride or hydrobromide salt of an amine (1 mmol) in anhydrous *N,N*-dimethylformamide (DMF) (2 mL) was added *N,N*-diisopropylethylamine (DIPEA) (2 mmol) to liberate the amine. After stirring at room temperature for 10 min, nonanoyl chloride (1 mmol) was added. The solution was stirred at room temperature for 6 to 24 h. After the reaction, water (40 mL) was added to the solution. The reaction mixture was transferred to a separating funnel and extracted with dichloromethane, CH_2_Cl_2_ (3×6 mL). The organic extracts were concentrated under reduced pressure to give a crude product. The crude product was then purified by silica gel column chromatography (using hexane/ethyl acetate, 2∶1 v/v, as eluent) or preparative HPLC (using the protocol set out above) to give the final product (Figure S10 in File S1).

### Synthetic Procedure for Analogues JC007, JC010 and JC017

To a solution of the hydrochloride or hydrobromide salt of an amine (1–2 mmol) in anhydrous dichloromethane, tetrahydrofuran or *N,N*-dimethylformamide (DMF) (2–4 mL) was added *N,N*-diisopropylethylamine (DIPEA) (1–2 mmol) to liberate the amine. After stirring at room temperature for 10 min, isocyanate (1–2 mmol) was added. The solution was stirred at room temperature for 6 to 24 h. After the reaction, water (20 mL) was added to the solution. The reaction mixture was transferred to a separating funnel and extracted with dichloromethane, CH_2_Cl_2_ (3×10 mL). The organic extracts were concentrated under reduced pressure to give a crude product, which was purified by silica gel column chromatography (using hexane/ethyl acetate, 2∶1 v/v, as eluent) or preparative HPLC (using the protocol set out above) to give the final product (Figure S11 in File S1)..

## Supporting Information

File S1 **Figures S1–S11.** Figure S1 in File S1: Table of LC-MS data for JC analogues. Figure S2 in File S1: NMR spectra for JC analogues. Figure S3 in File S1: Zebrafish acute toxicity and developmental study data. Figure S4 in File S1: Normal human astrocytes (Lonza) were treated with JC005, JC010, JC011 and JC017 at 20 µM final concentration for 12 hrs following which the Resazurin assay was used to determine cell viability. Cell death values are normalized to untreated controls and reported as mean ± S.D. of three independent experiments (n = 3). Figure S5 in File S1: Time-course cell viability study to determine kinetics of JC011 mediated cell death in 3 PSC lines. Maximum cell death of approximately 96% is attained for both BGO1V and H9 following a 36 hr incubation with 20 µM JC011. Cell death values are normalized to untreated controls and reported as mean ± S.D. of three independent experiments (n = 3). Figure S6 in File S1: Cell viability analysis for JC011 (20 µM, 12 hrs) treated HepG2, HeLa, WI-38 and normal human keratinocytes. Resultant cell viability as determined by Resazurin analysis remained high at >97.5% for all cell lines. Cell death values are normalized to untreated controls and reported as mean ± S.D. of three independent experiments (n = 3). Figure S7 in File S1: Tra-1-60 and SSEA-4 immunomarker FACS analysis for BGO1V, H9 and iPSC-foreskin-1. All PSC lines were >95% positive for both Tra-1-60 and SSEA-4 stem cell-specific antigens (n = 3). Figure S8 in File S1: Non-specific ER stress inducer DTT does not induce cell death in BGO1V. PSC-cytotoxicity in BGO1V cells could not be replicated with DTT treatment confirming that JC011 mediated PSC-cytotoxicity is a property specific to the JC molecule series. Cell death values are normalized to untreated controls and reported as mean ± S.D. of three independent experiments (n = 3), * = P<0.05. Figure S9 in File S1: Surrogate ROS levels in NCCIT following ATF4 and DDIT3 siRNA knockdown. ATF4 knockdown resulted in a recovery of ROS levels in JC011 (20 µM) treated NCCIT cells comparable to untreated controls. DDIT3 knockdown resulted in no significant recovery in ROS levels (n = 3). Figure S10 in File S1: Synthetic Procedure for Analogues JC005, JC011, JC040, JC048-050. Figure S11 in File S1: Synthetic Procedure for Analogues JC007, JC010 and JC017.
(DOCX)
