# Supplementary material for: A New Class of Pluripotent Stem Cell Cytotoxic Small Molecules
Source: PLoS One. 2014 Mar 19;9(3):e85039. doi: 10.1371/journal.pone.0085039 (PMC3960094; doi:10.1371/journal.pone.0085039)
Supplement: File S1 — Figures S1–S11. Figure S1 in File S1: Table of LC-MS data for JC analogues. Figure S2 in File S1: NMR spectra for JC analogues. Figure S3 in File S1: Zebrafish acute toxicity and developmental study data. Figure S4 in File S1: Normal human astrocytes (Lonza) were treated with JC005, JC010, JC011 and JC017 at 20 µM final concentration for 12 hrs following which the Resazurin assay was used to determine cell viability. Cell death values are normalized to untreated controls and reported as mean ± S.D. of three independent experiments (n = 3). Figure S5 in File S1: Time-course cell viability study to determine kinetics of JC011 mediated cell death in 3 PSC lines. Maximum cell death of approximately 96% is attained for both BGO1V and H9 following a 36 hr incubation with 20 µM JC011. Cell death values are normalized to untreated controls and reported as mean ± S.D. of three independent experiments (n = 3). Figure S6 in File S1: Cell viability analysis for JC011 (20 µM, 12 hrs) treated HepG2, HeLa, WI-38 and normal human keratinocytes. Resultant cell viability as determined by Resazurin analysis remained high at >97.5% for all cell lines. Cell death values are normalized to untreated controls and reported as mean ± S.D. of three independent experiments (n = 3). Figure S7 in File S1: Tra-1-60 and SSEA-4 immunomarker FACS analysis for BGO1V, H9 and iPSC-foreskin-1. All PSC lines were >95% positive for both Tra-1-60 and SSEA-4 stem cell-specific antigens (n = 3). Figure S8 in File S1: Non-specific ER stress inducer DTT does not induce cell death in BGO1V. PSC-cytotoxicity in BGO1V cells could not be replicated with DTT treatment confirming that JC011 mediated PSC-cytotoxicity is a property specific to the JC molecule series. Cell death values are normalized to untreated controls and reported as mean ± S.D. of three independent experiments (n = 3), * = P<0.05. Figure S9 in File S1: Surrogate ROS levels in NCCIT following ATF4 and DDIT3 siRNA knockdown. ATF4 knockdown resulted [file pone.0085039.s001.docx]

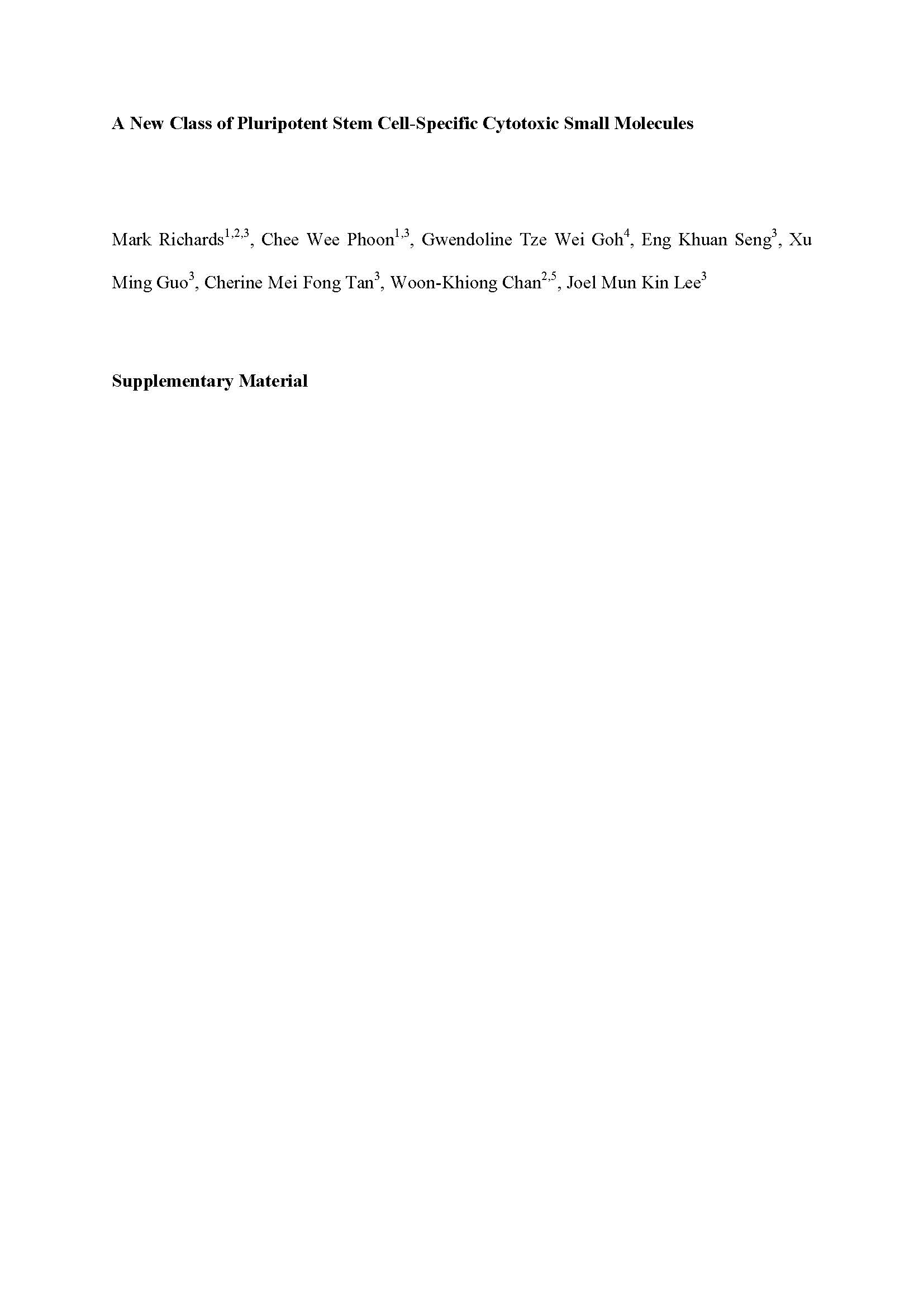


File S1


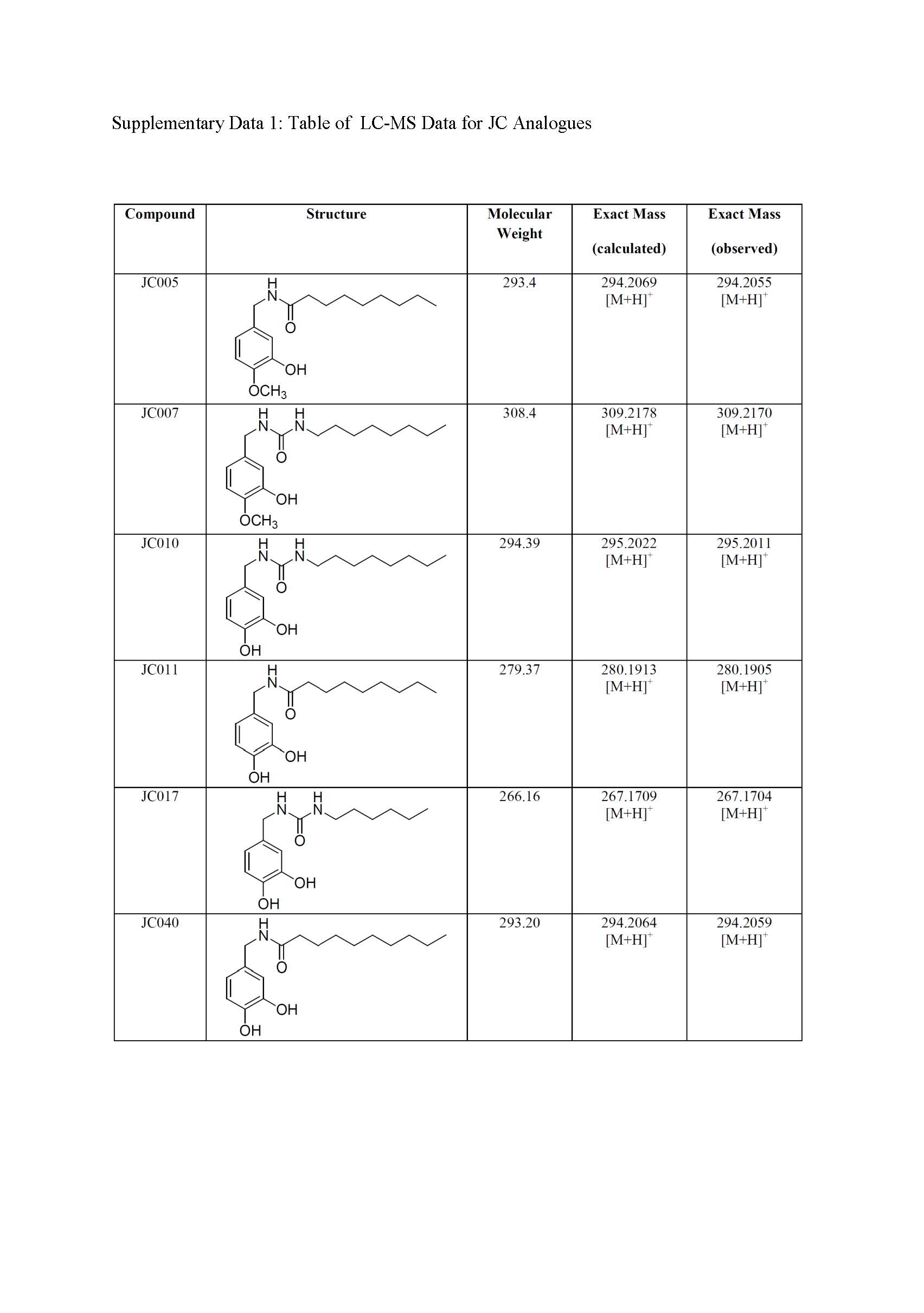

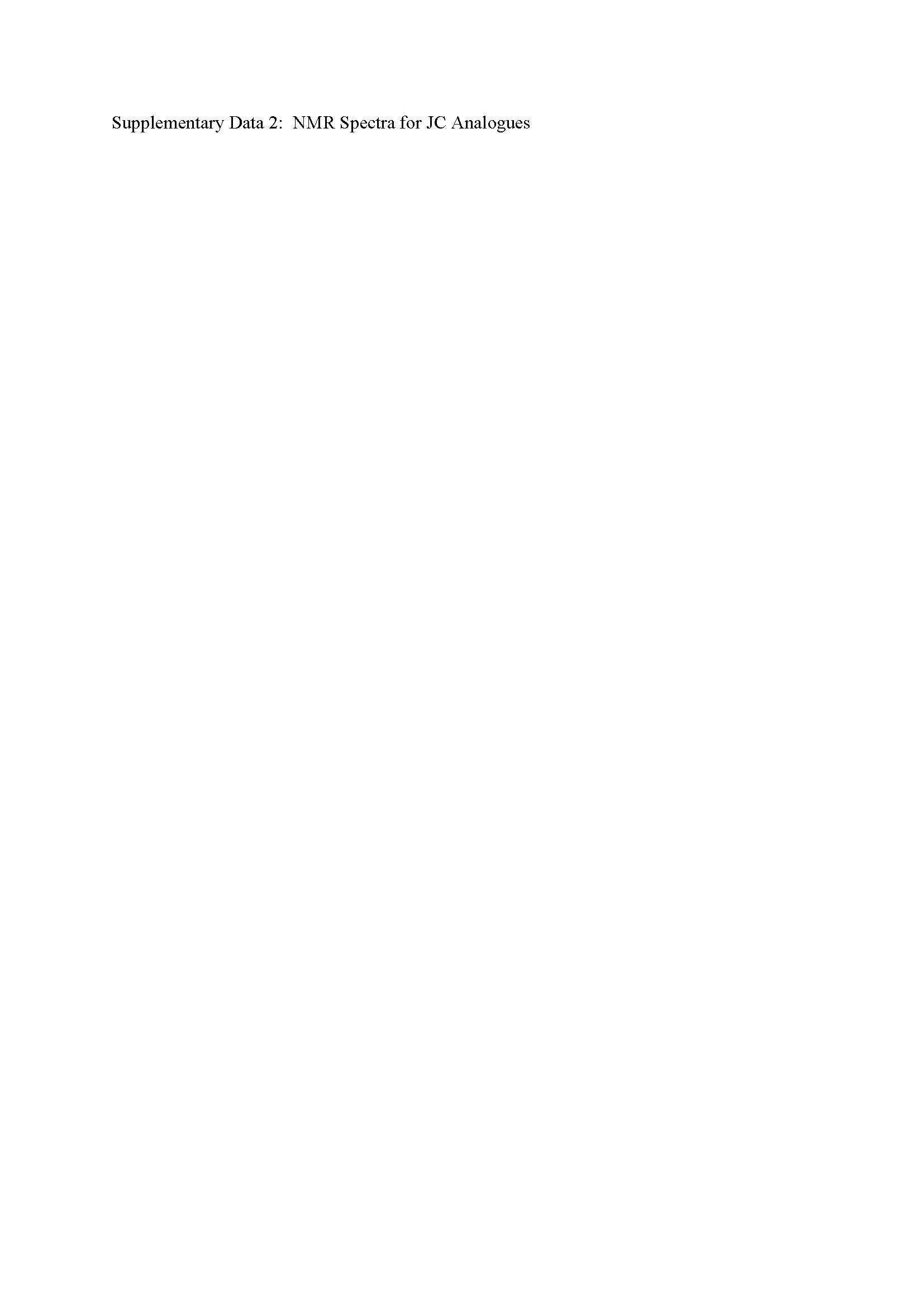

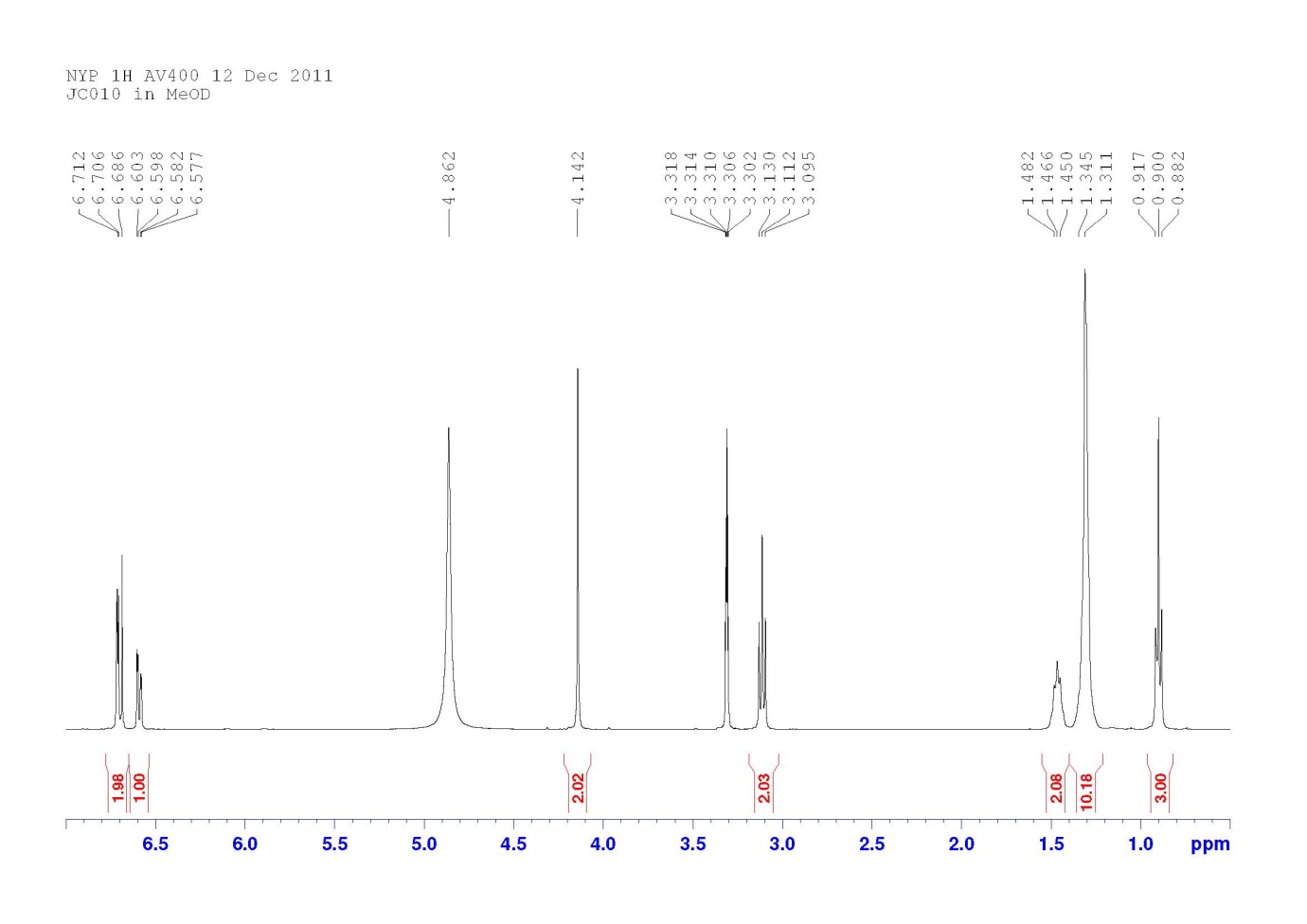

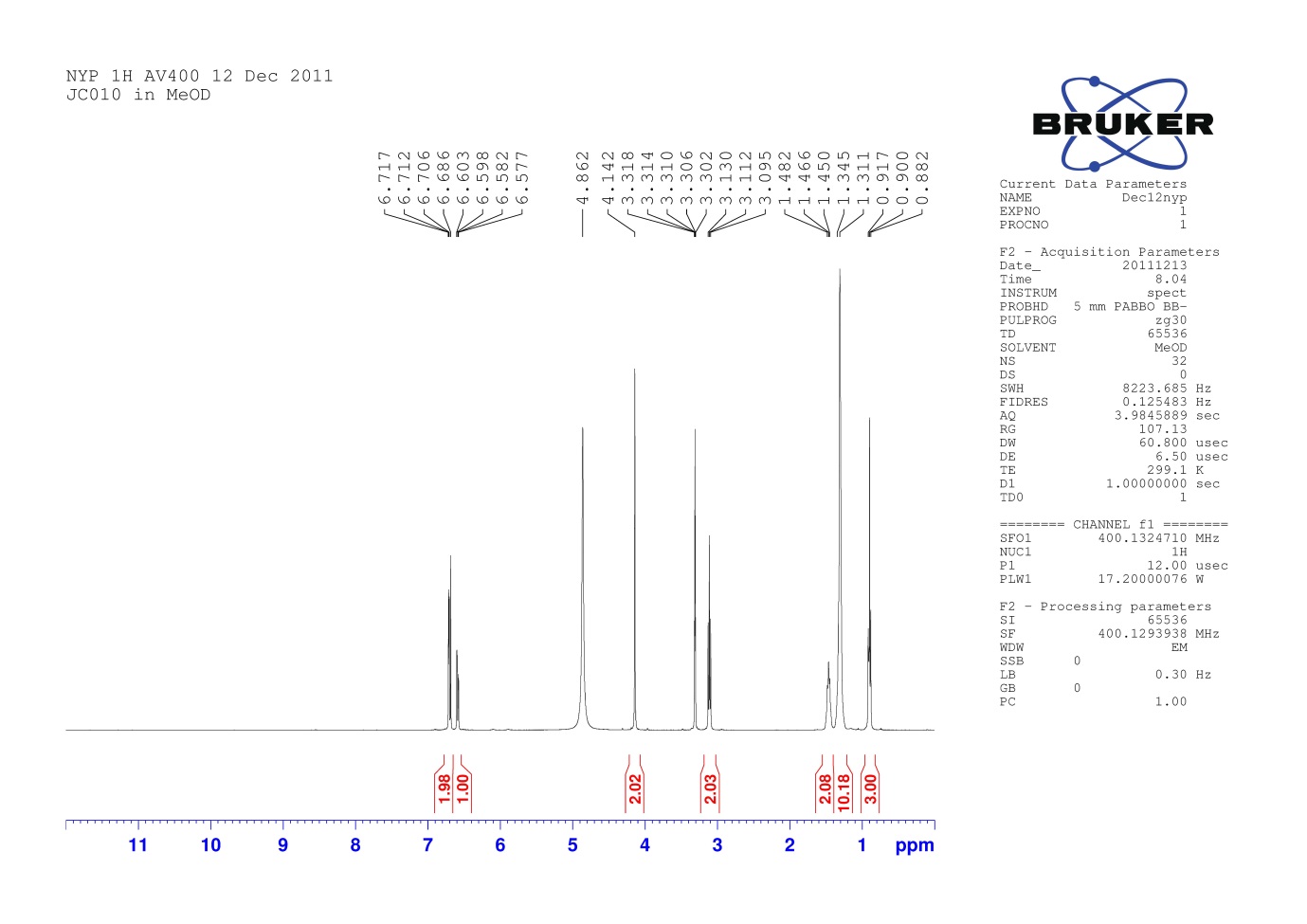

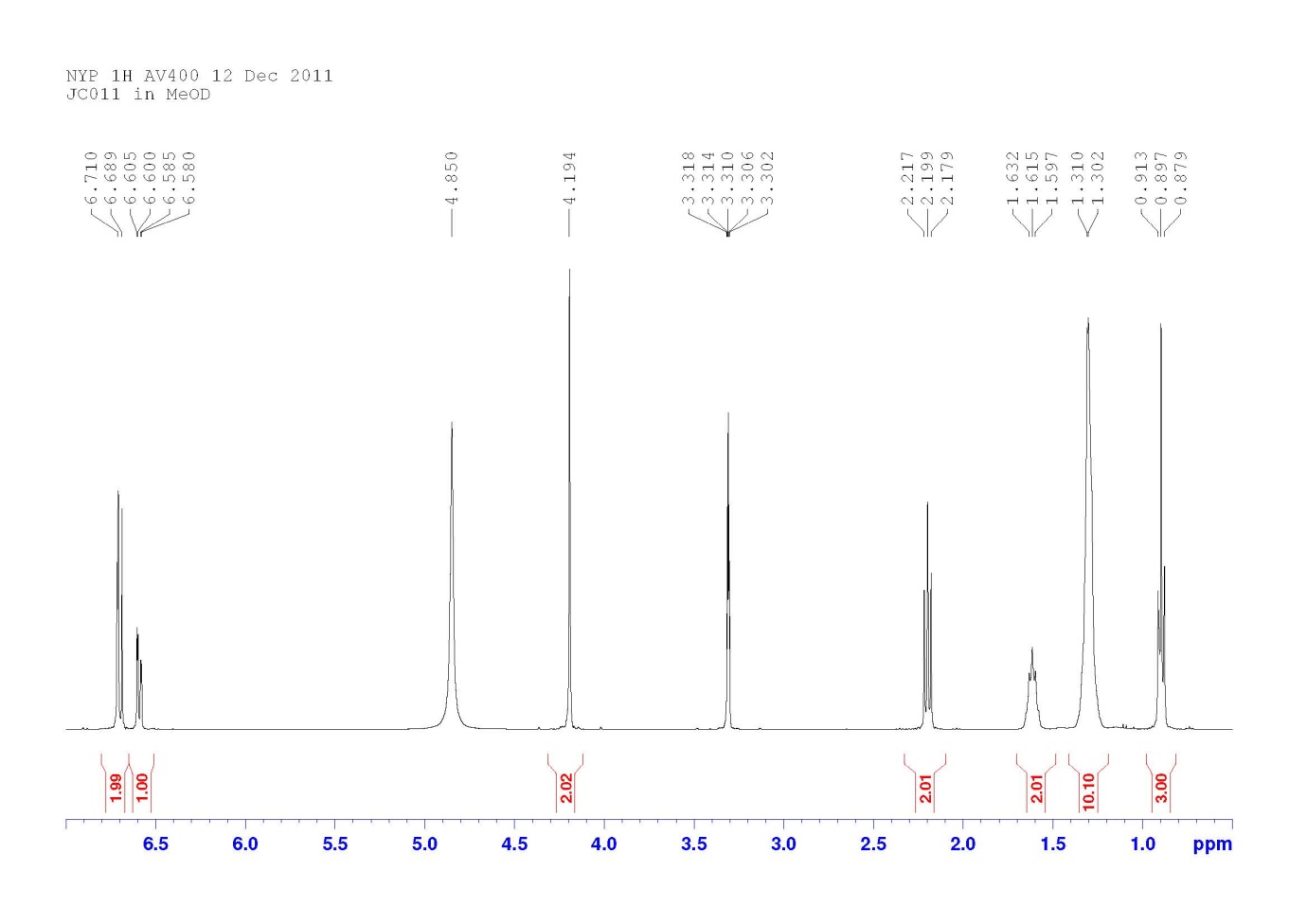

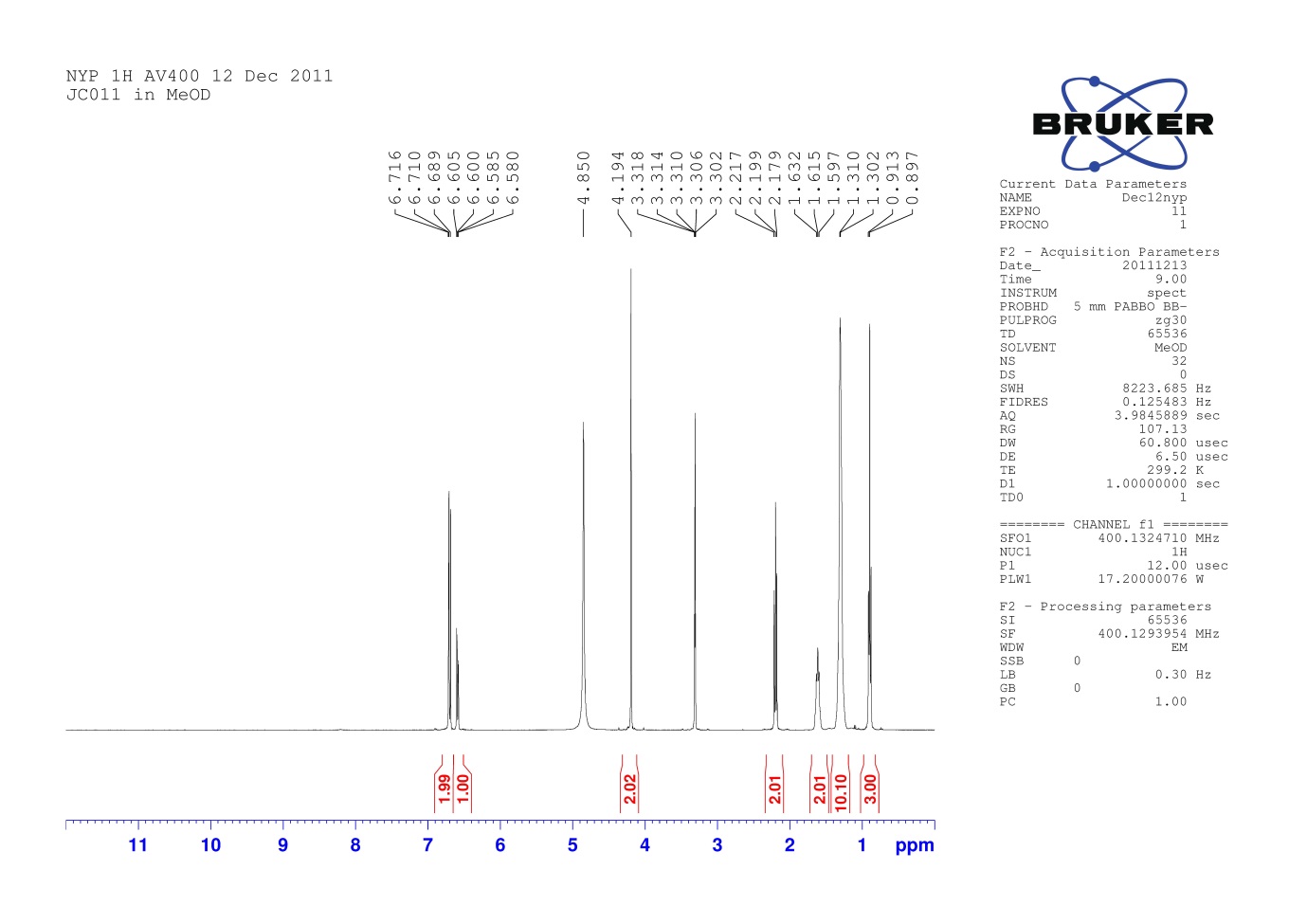

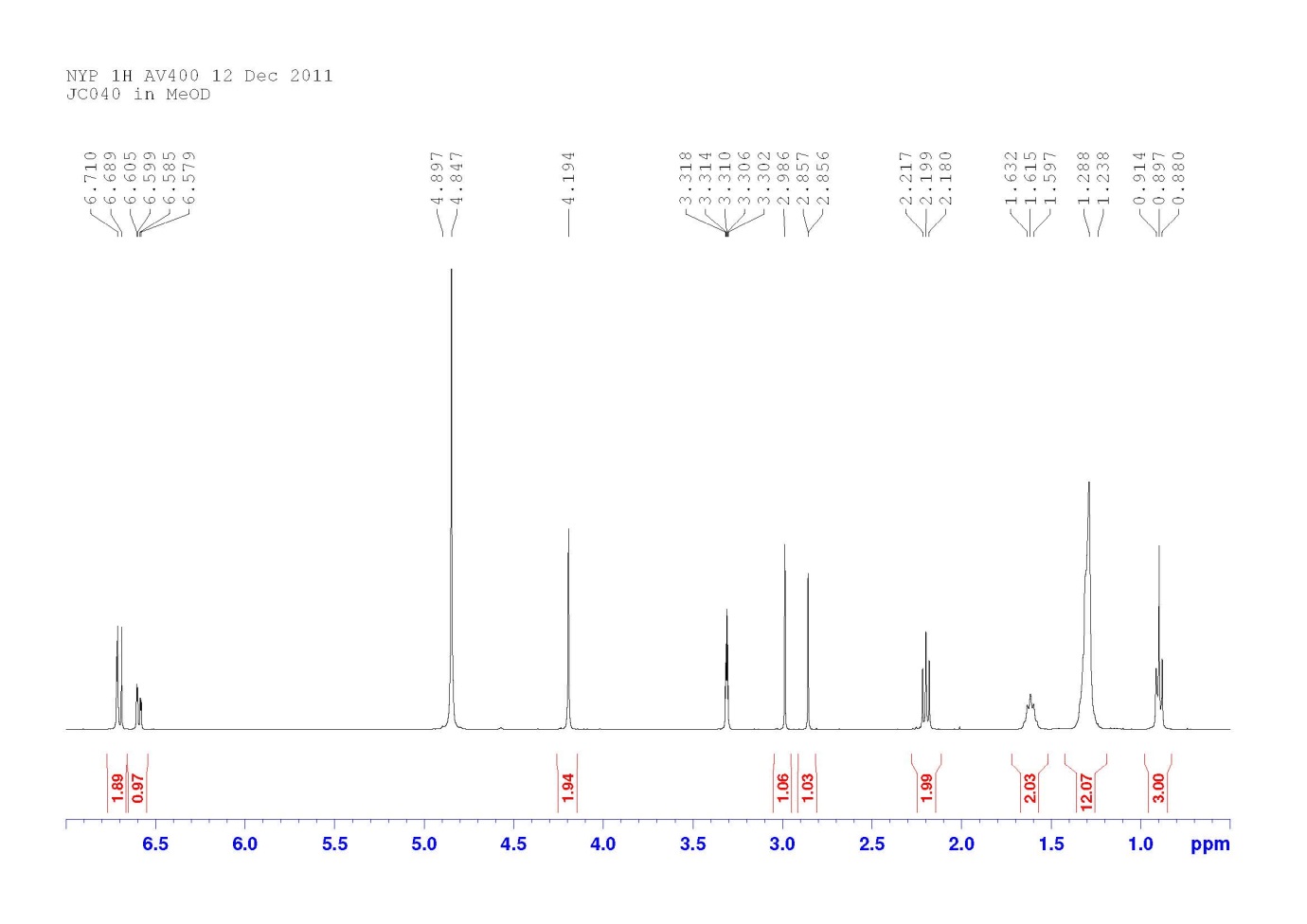

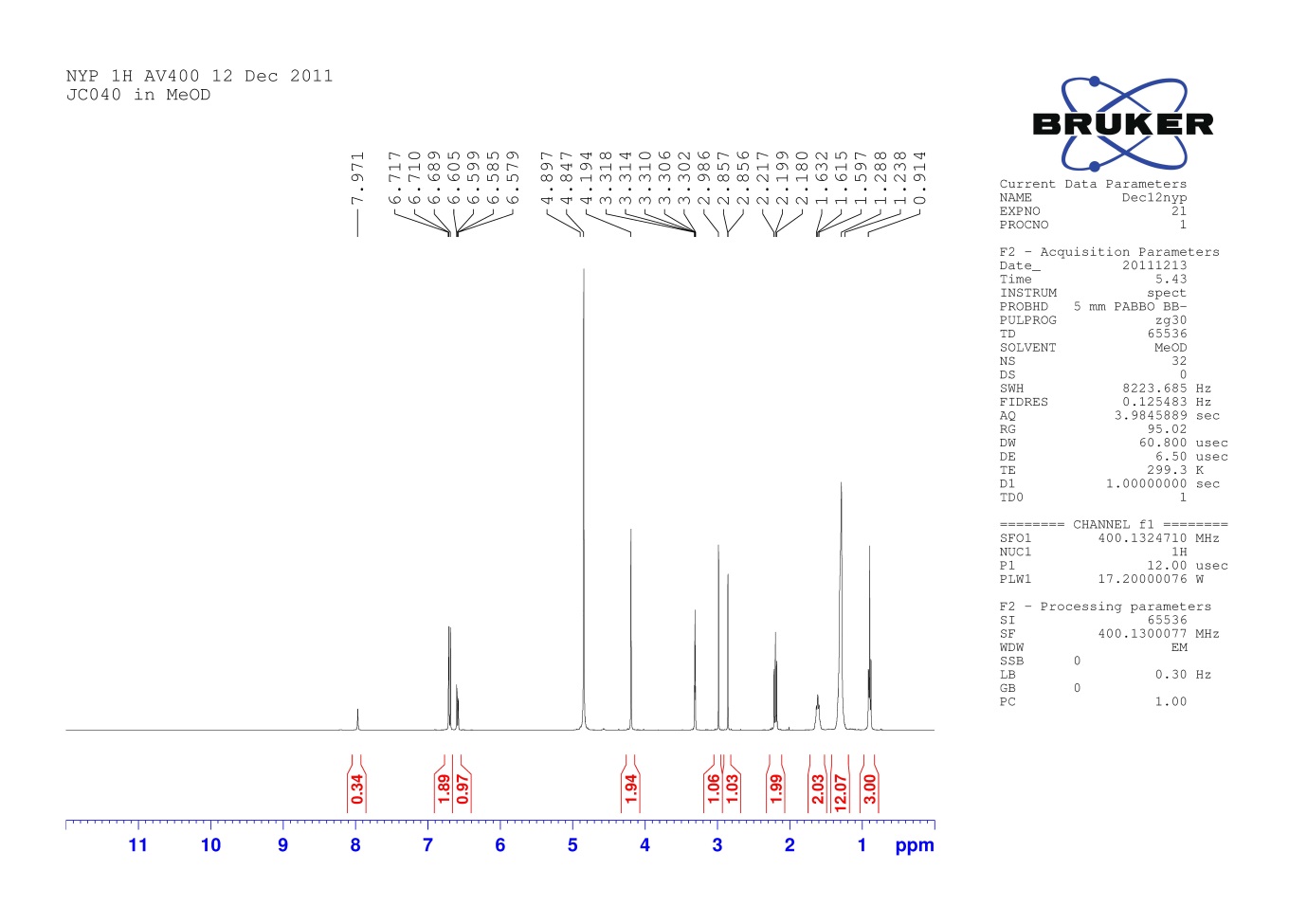

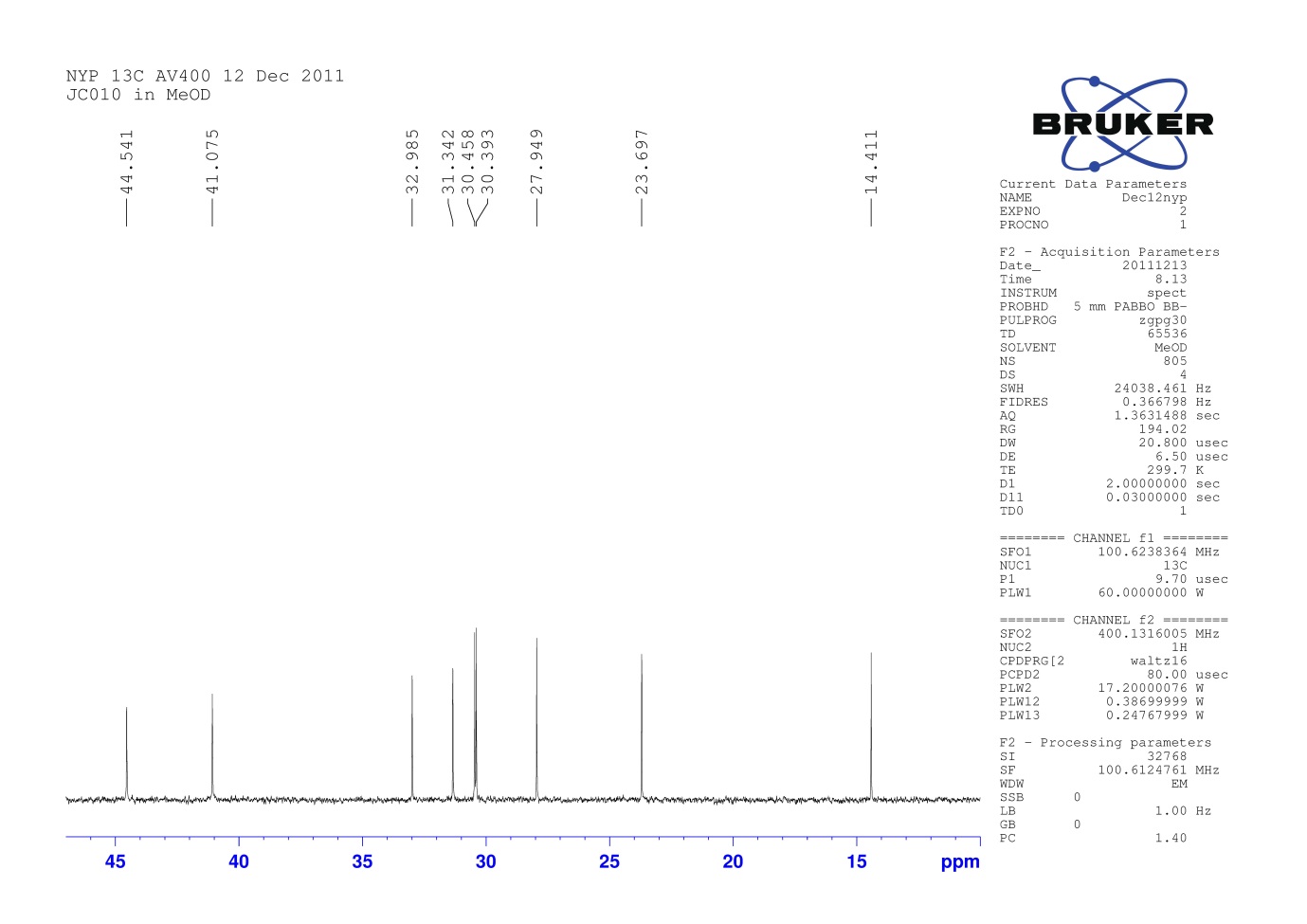

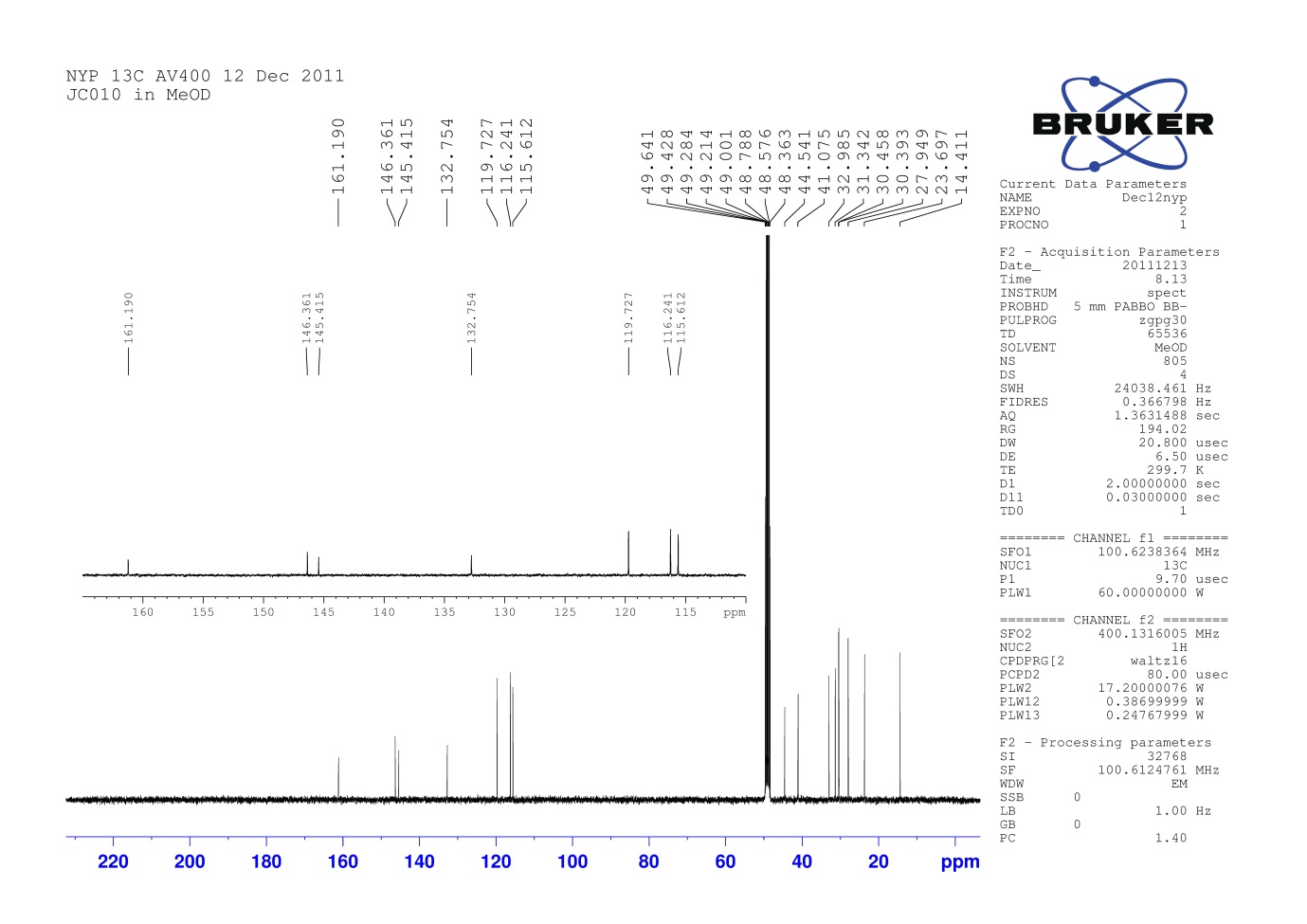

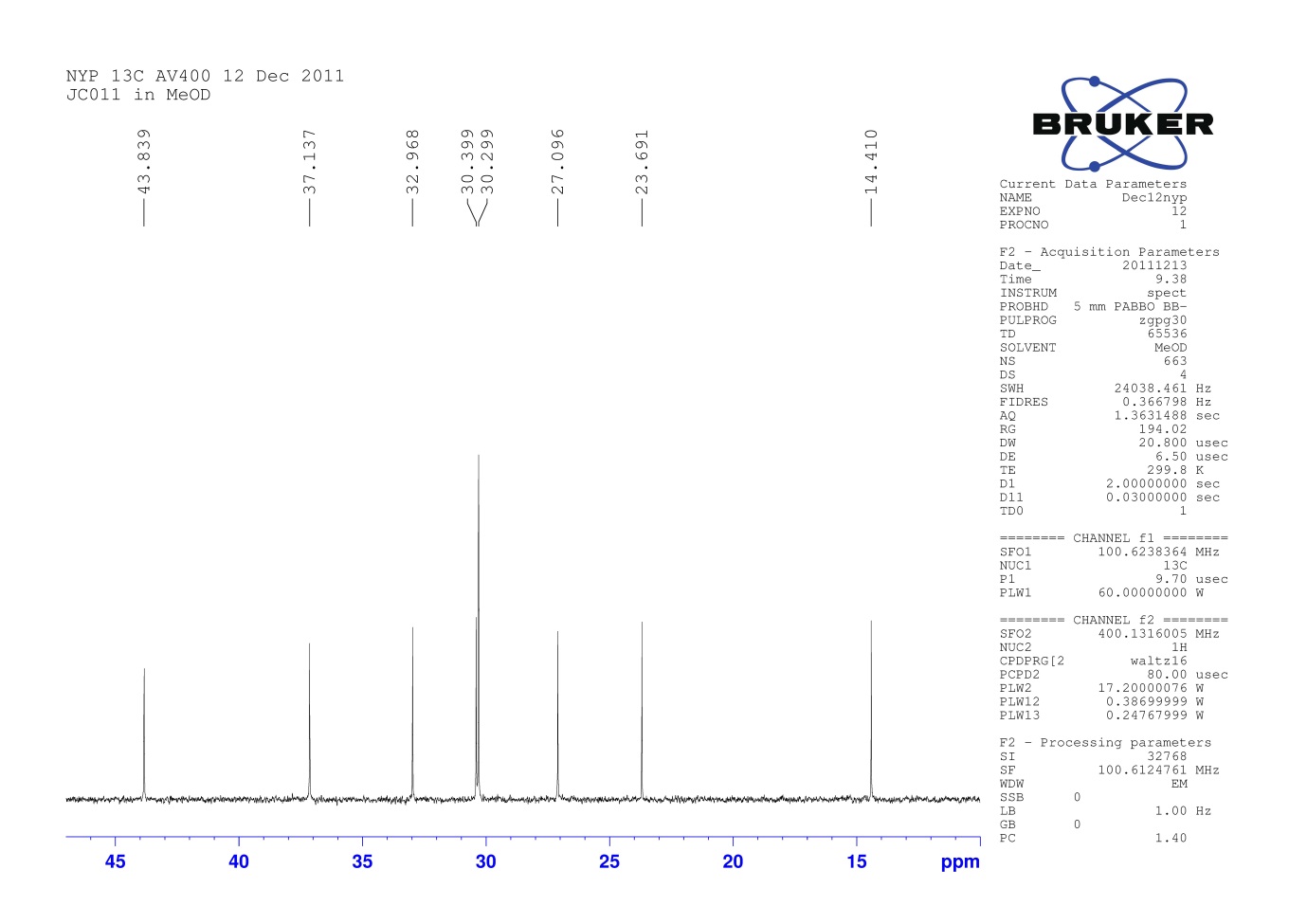

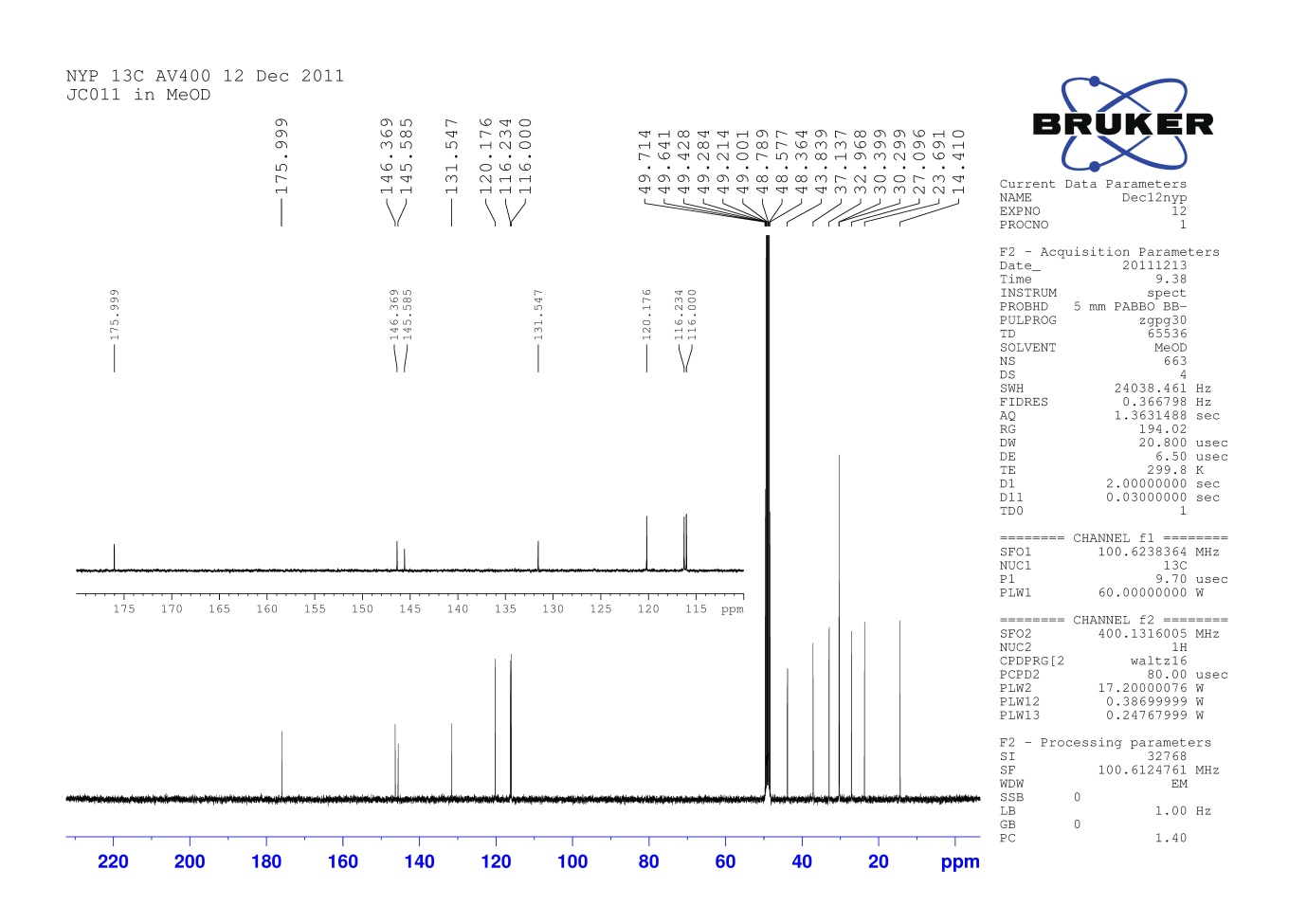

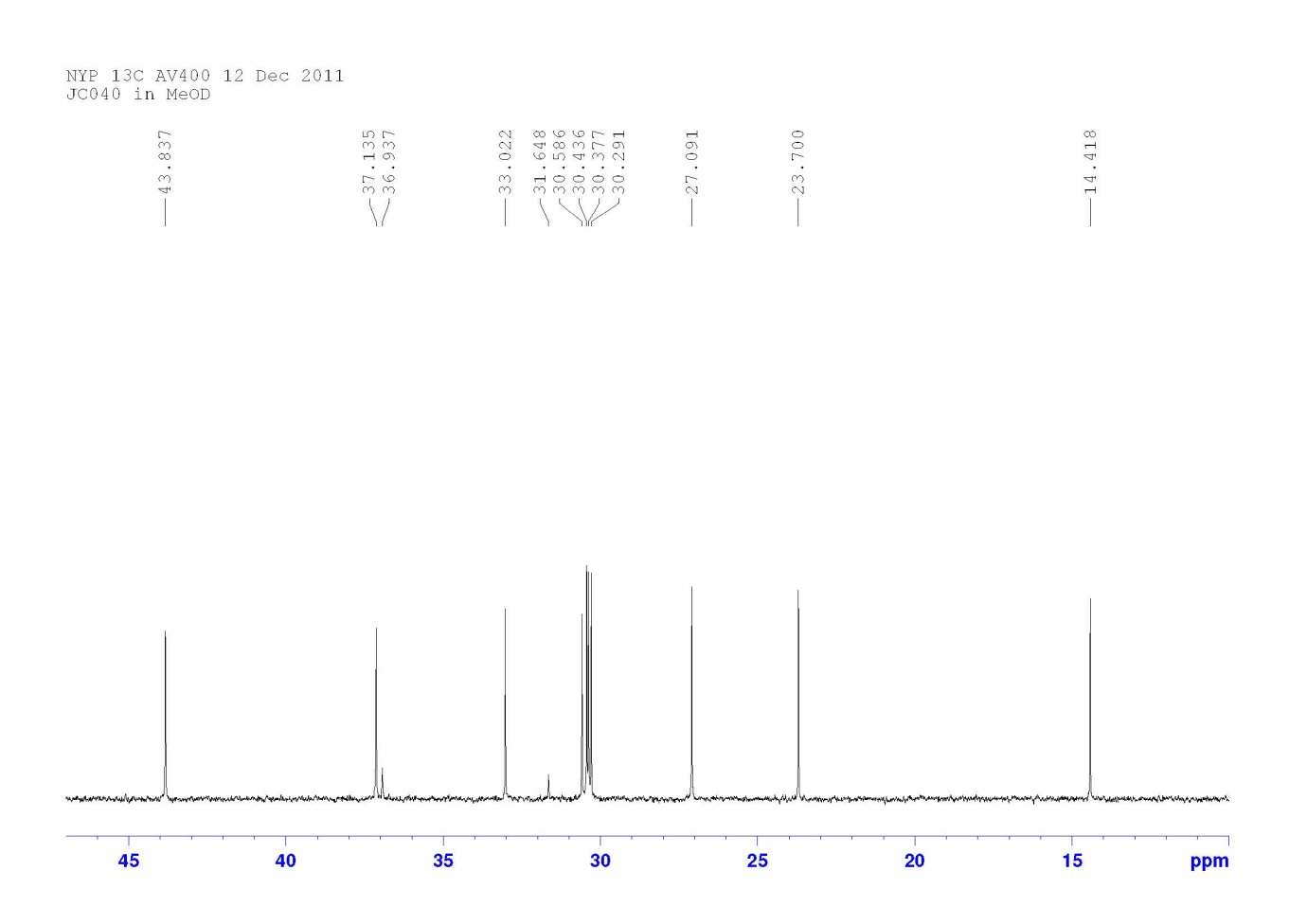

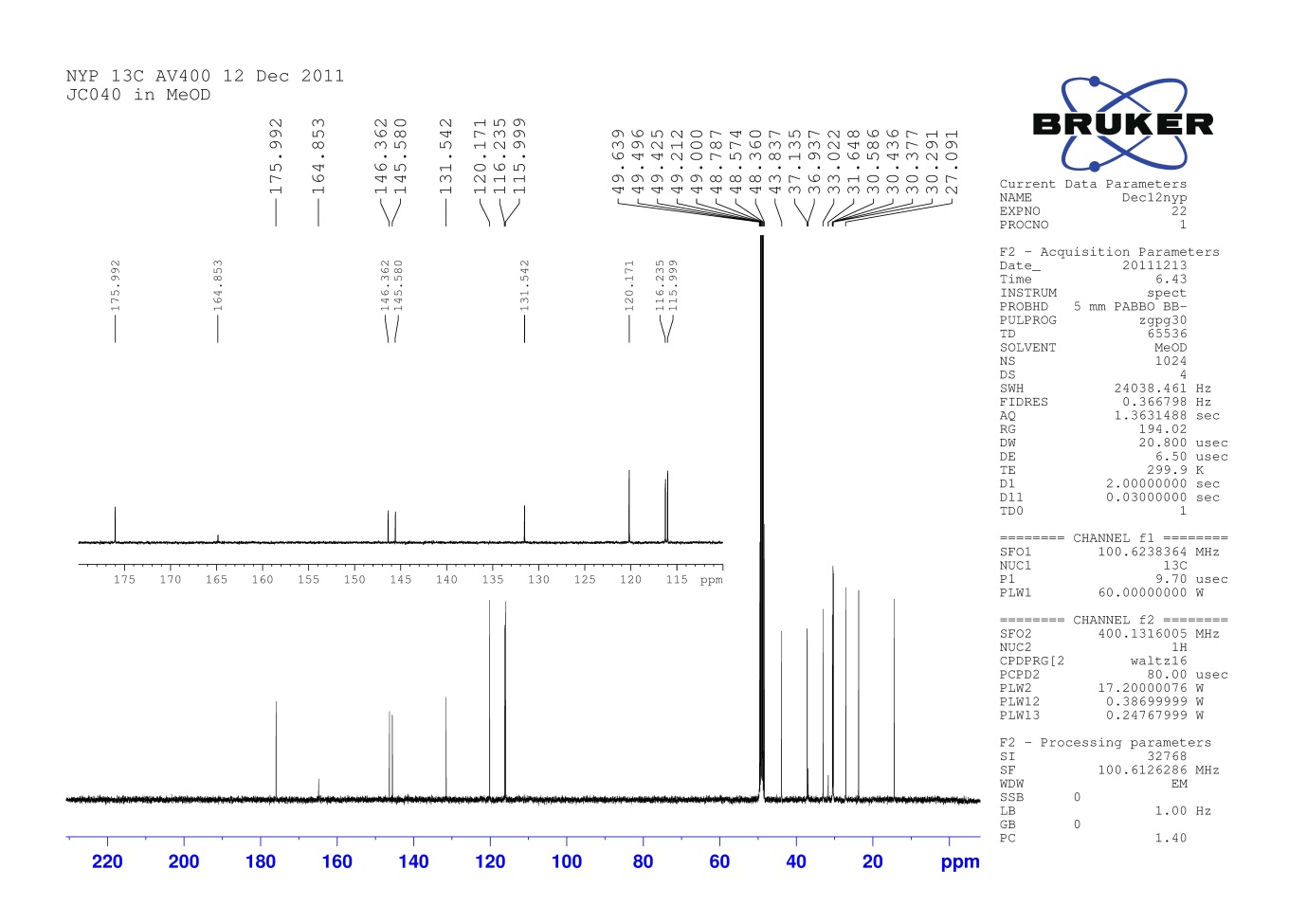

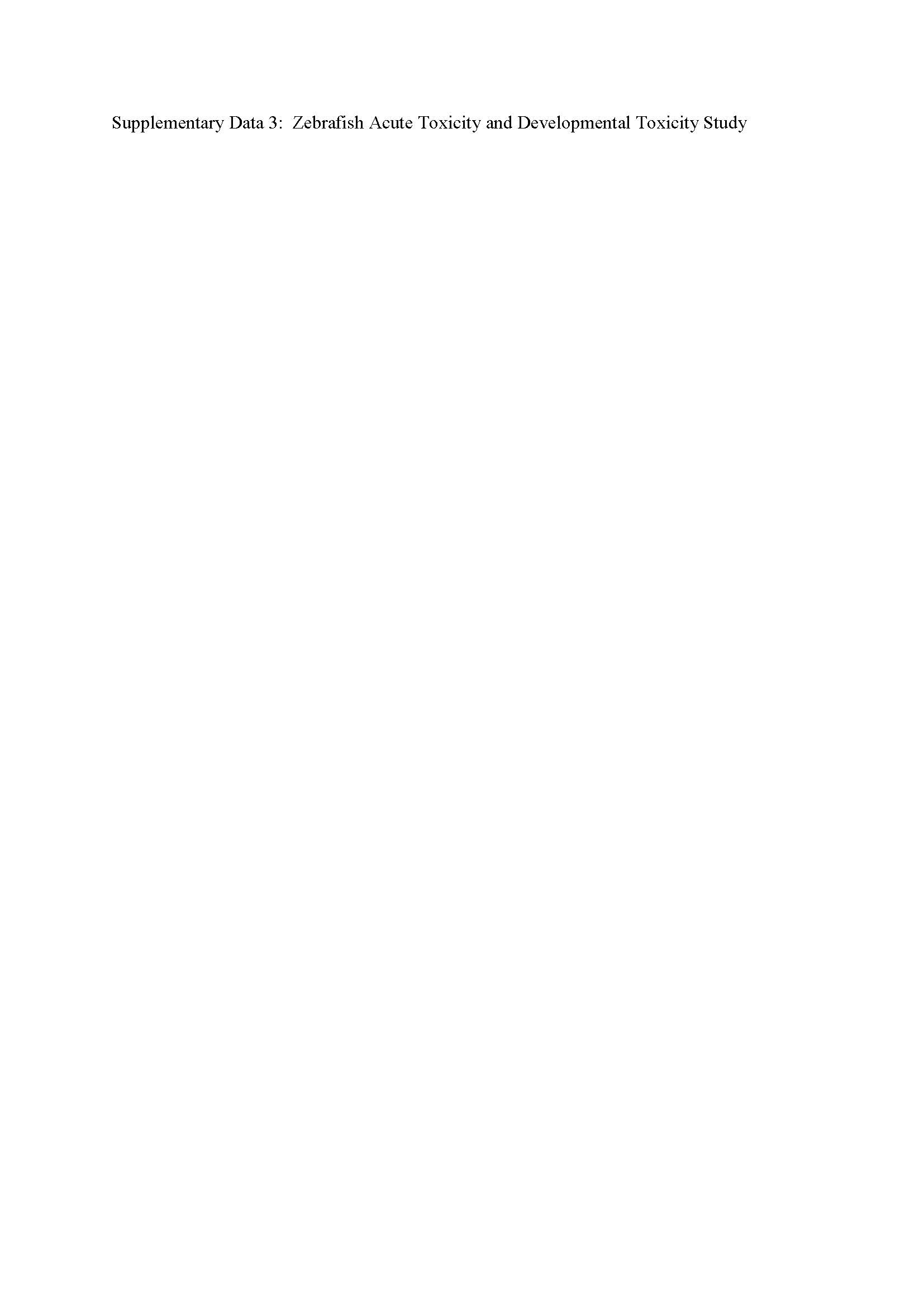

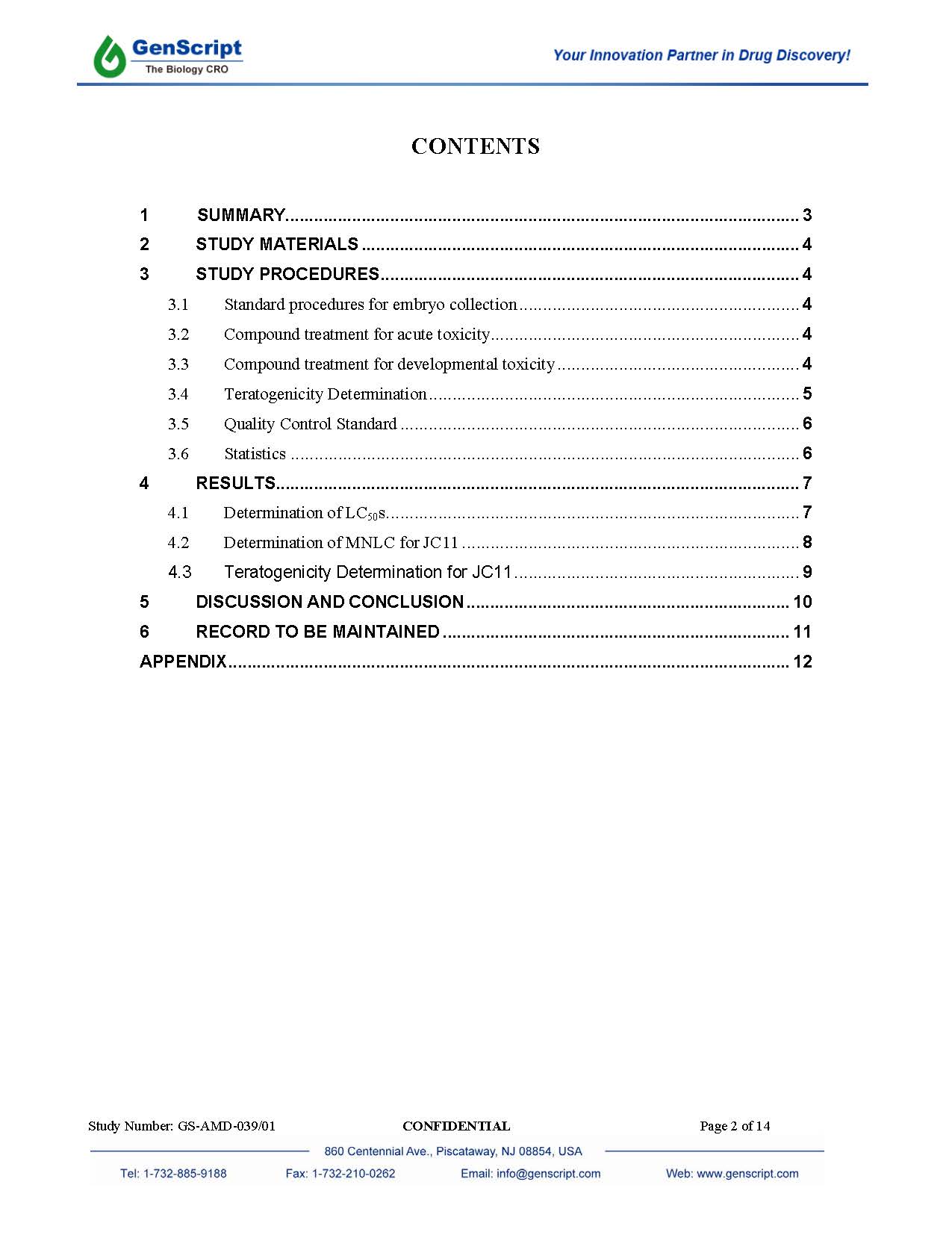

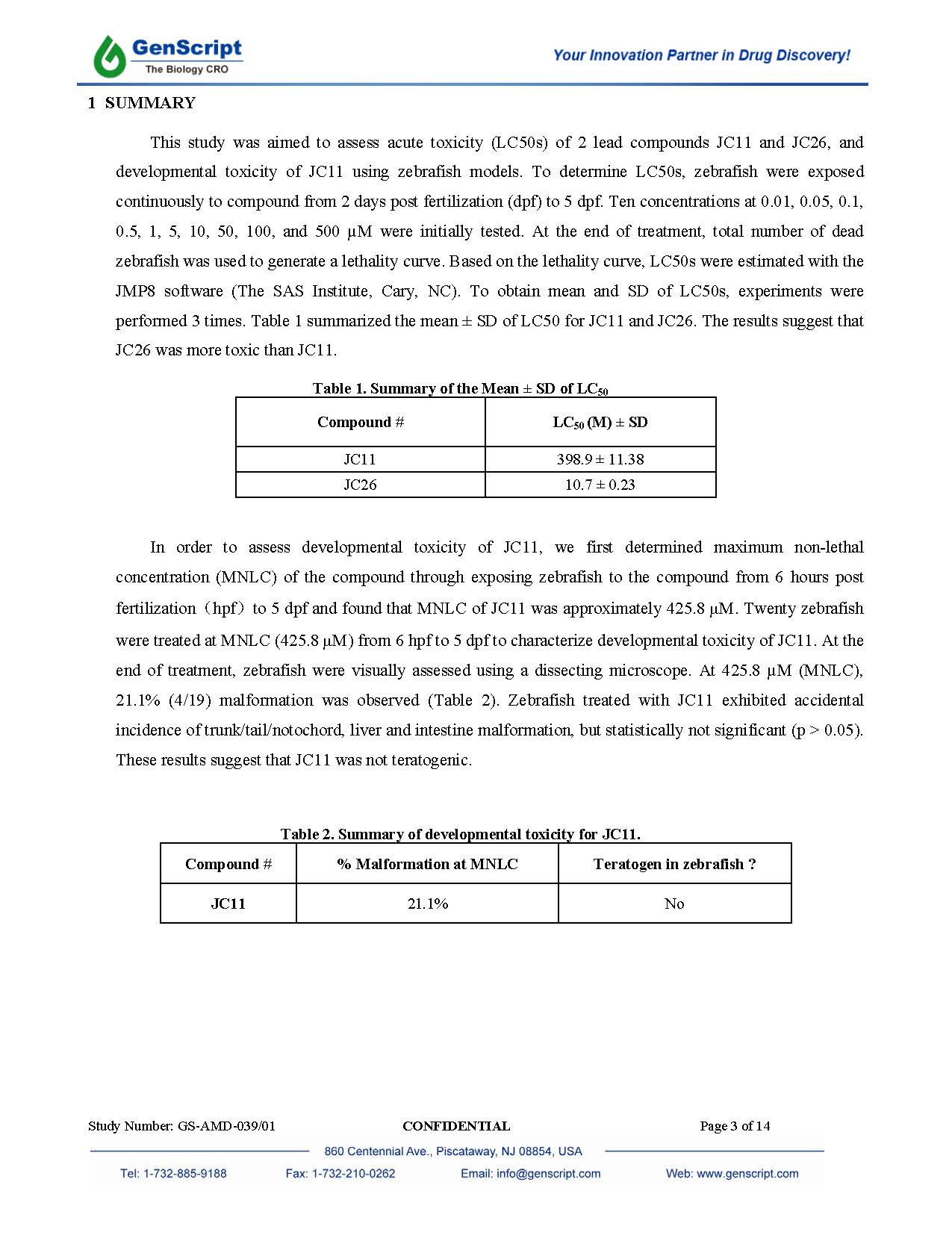

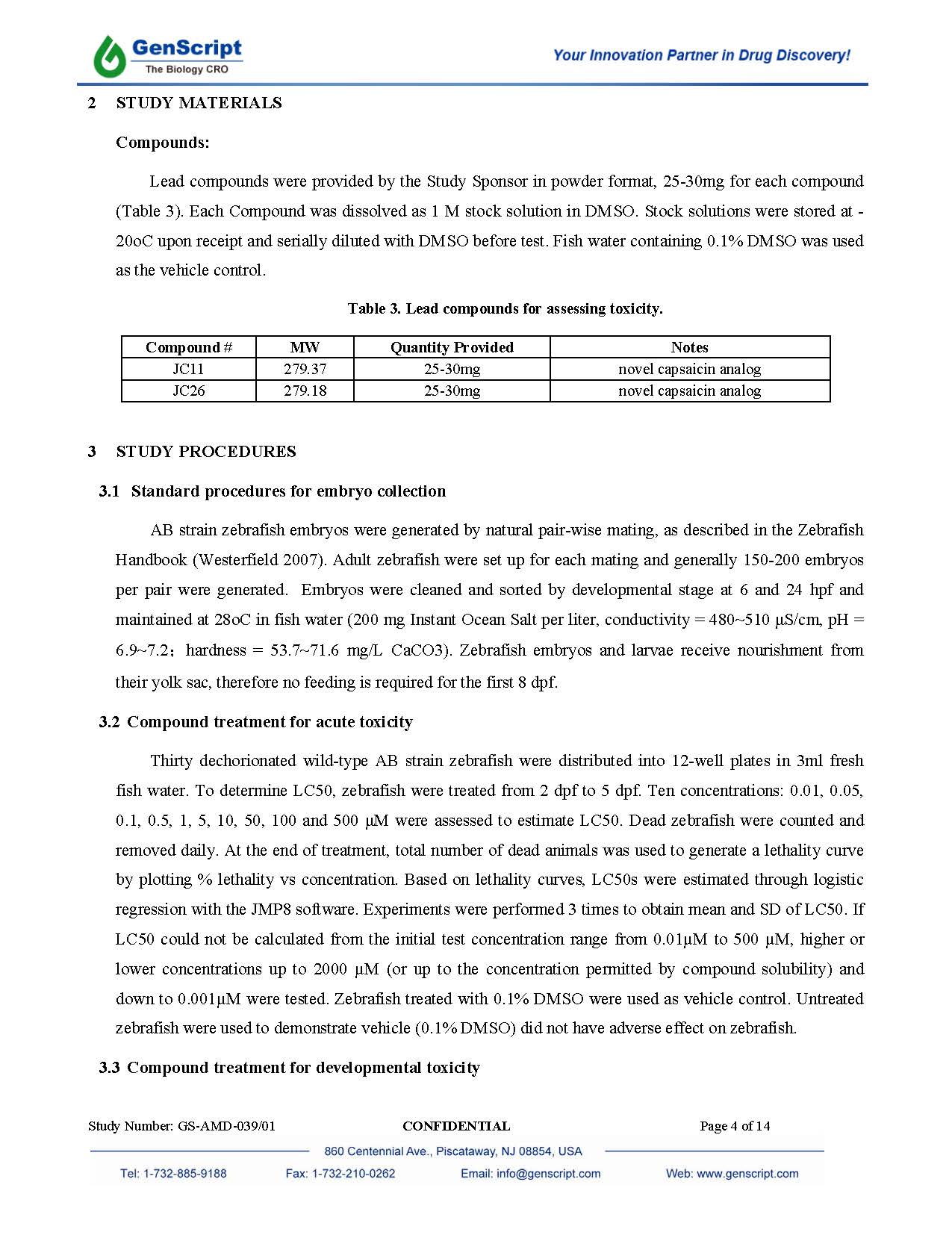

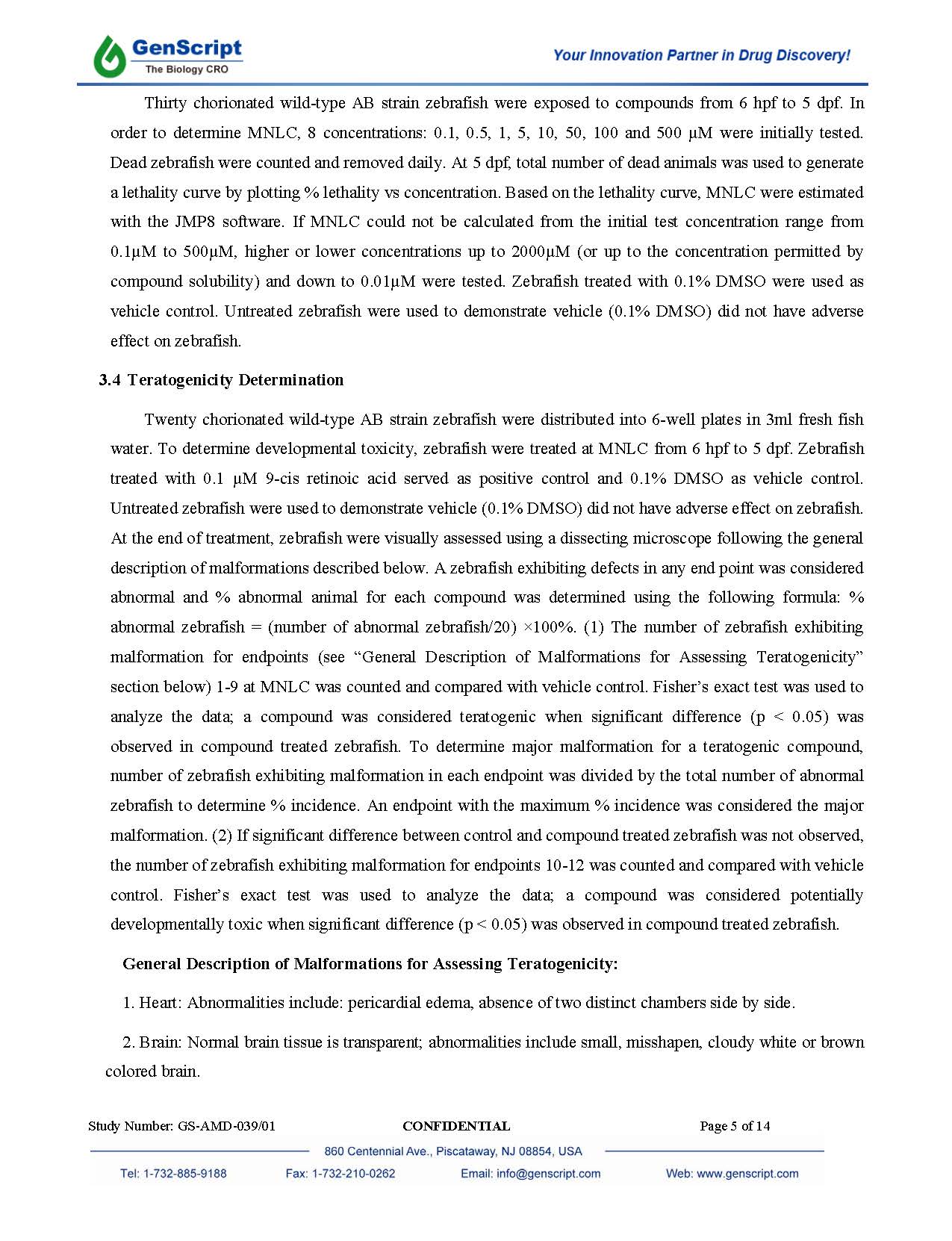

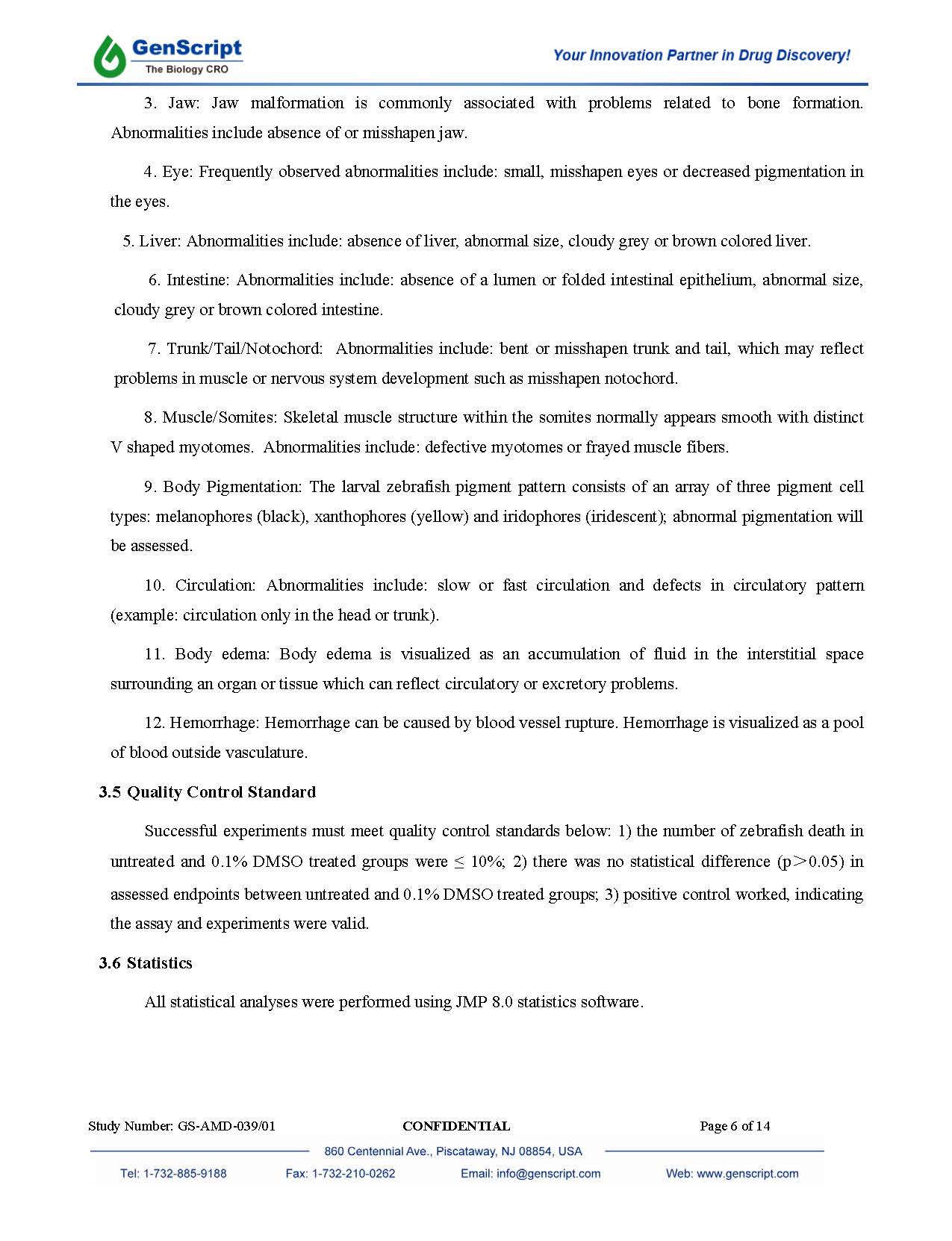

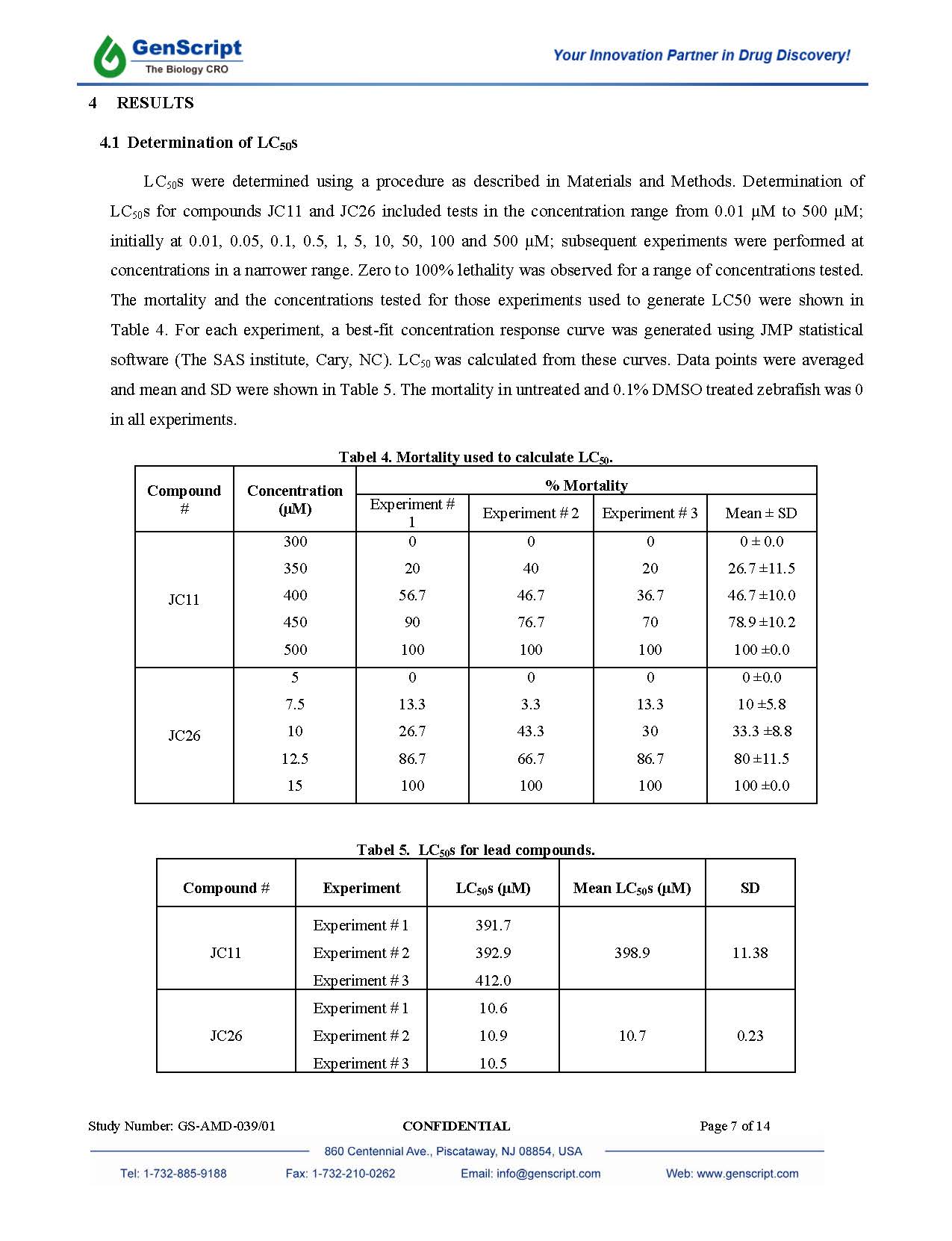

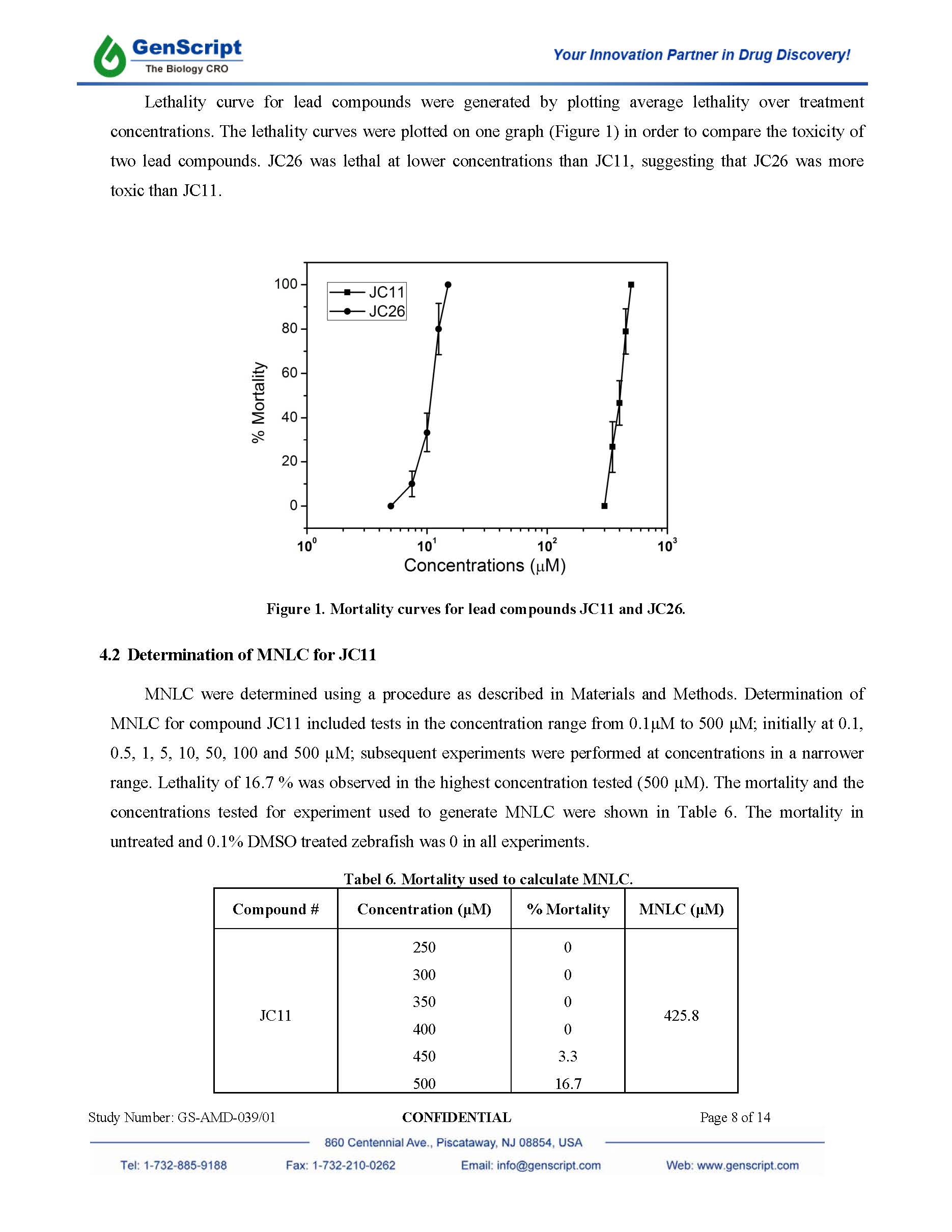

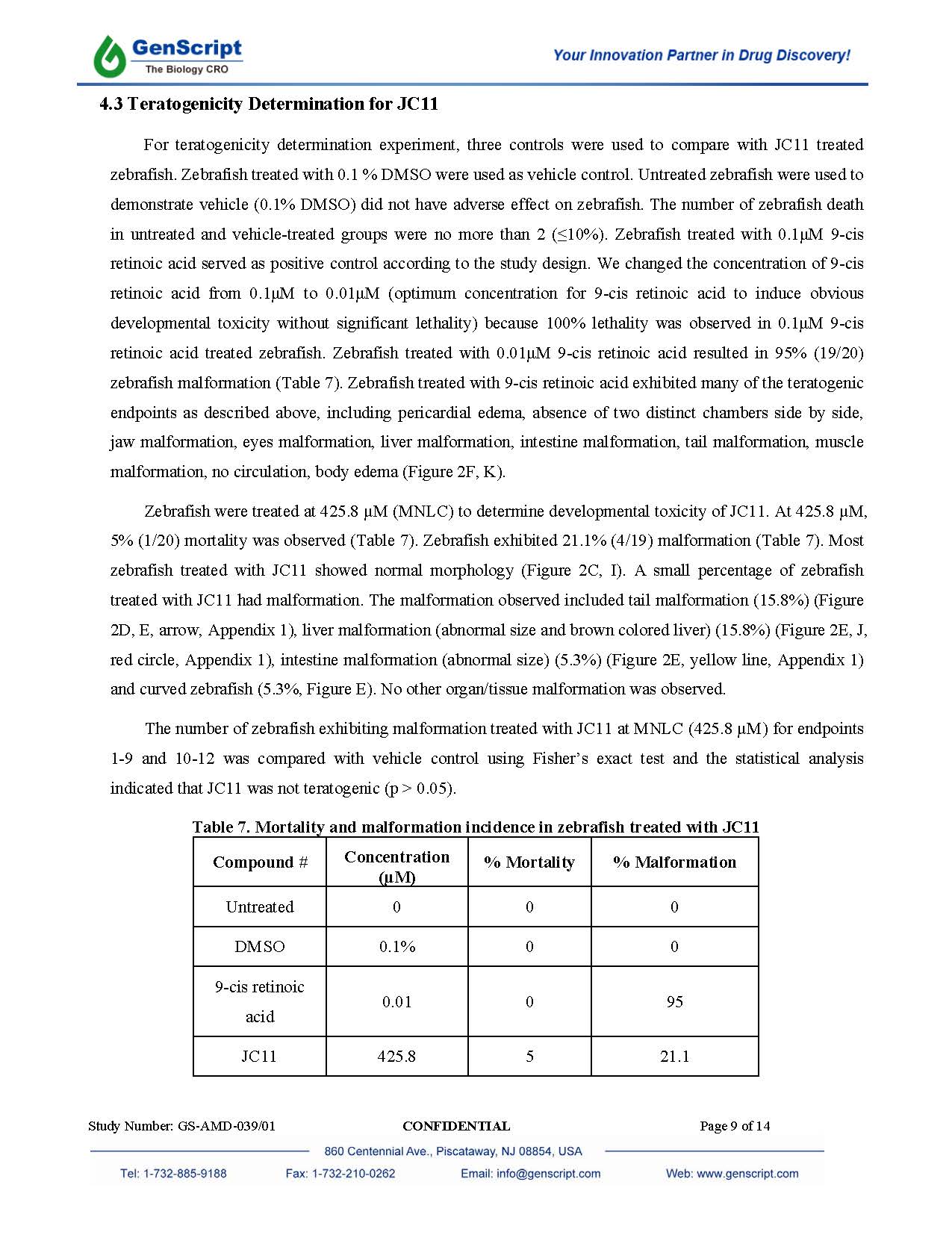

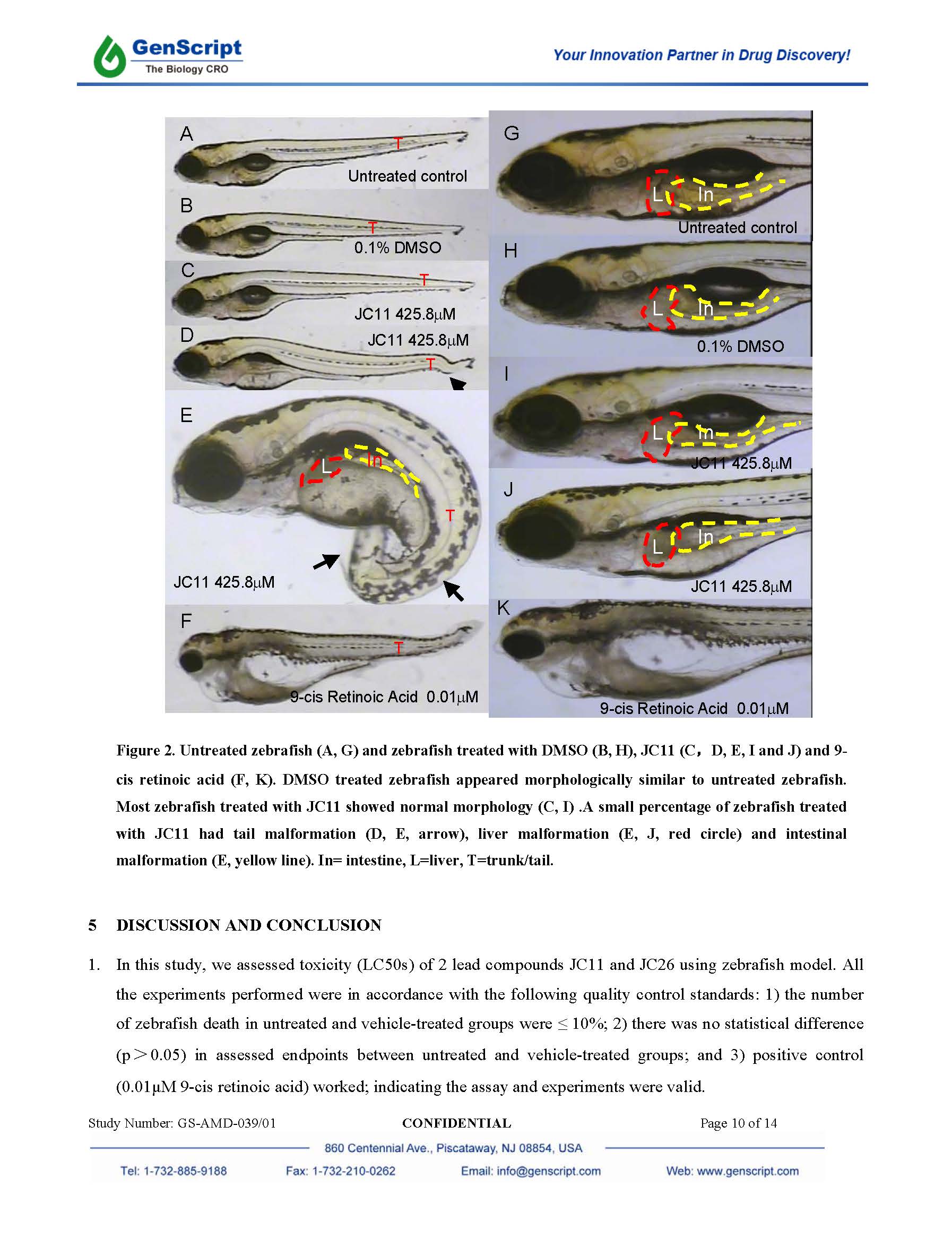

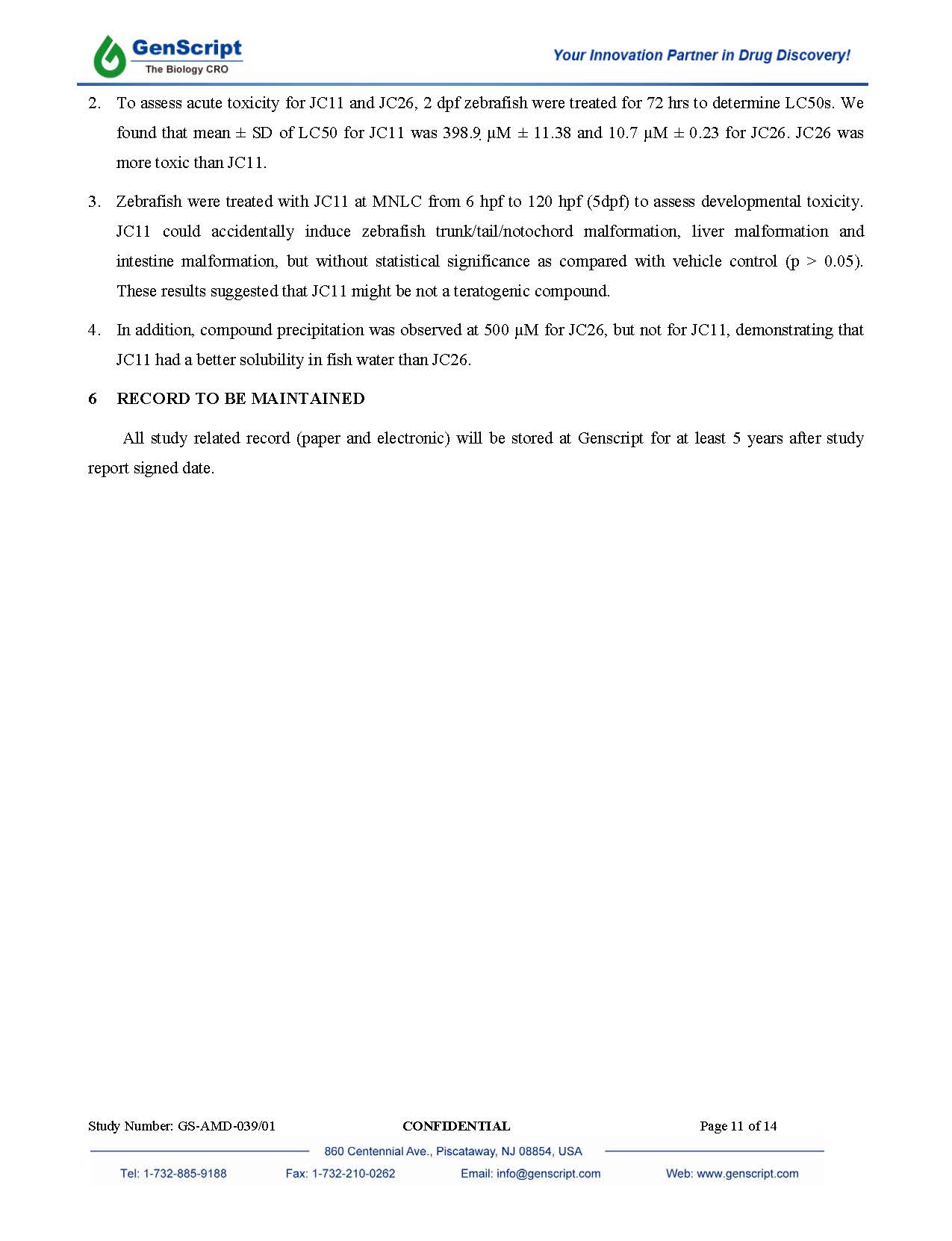

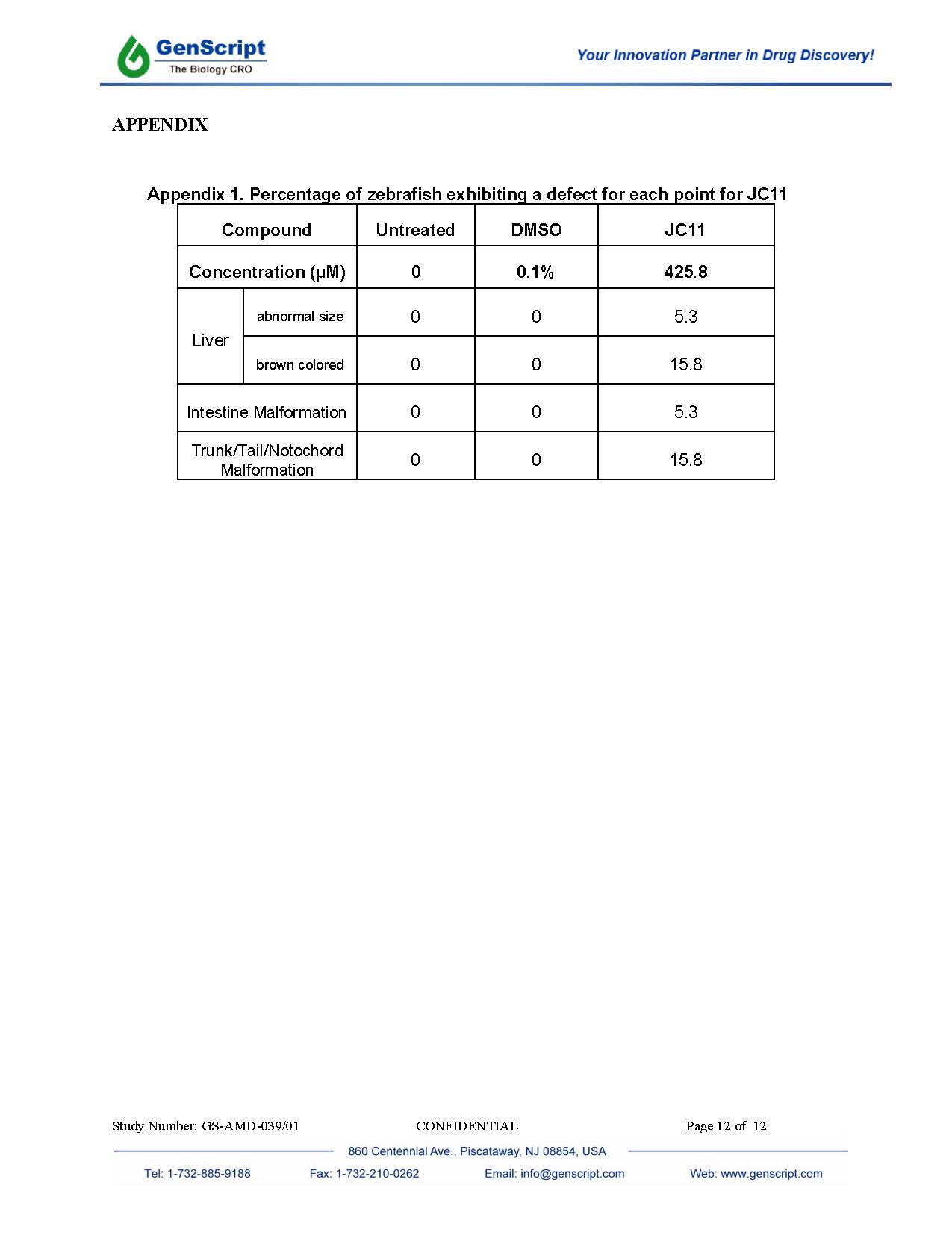


Figure S1 in File S1: Table of LC-MS data for JC analogues.

Figure S2 in File S1: NMR spectra for JC analogues.

Figure S3 in File S1: Zebrafish acute toxicity and developmental study data.

Figure 4 in File S1: JC Analogues are not cytotoxic to normal human astrocytes


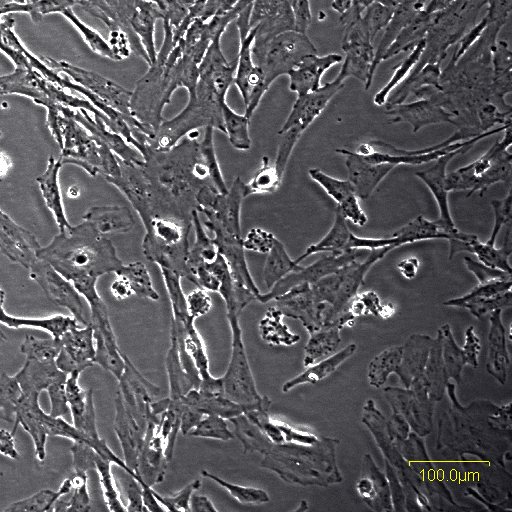

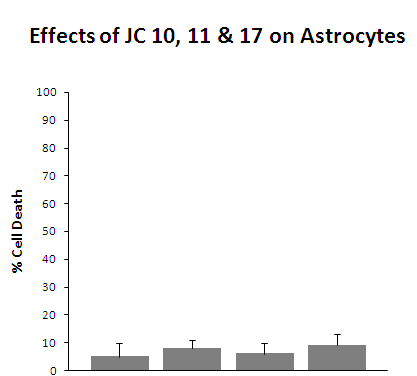


JC010

JC005

JC011

JC017

Figure 5 in File S1: Time course cell viability data for JC011 (20 μM) treated

BGO1V, H9 & iPS-Foreskin-1


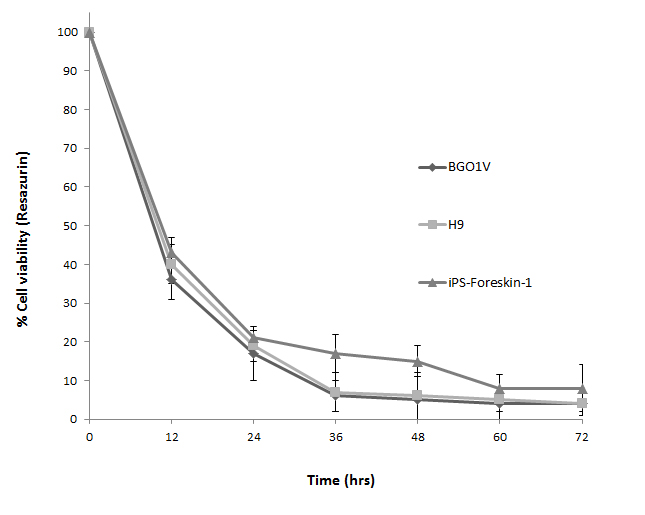


Figure 6 in File S1: Cell viability data for JC011 (20 μM) treated Hela, HepG2, WI-38 & Keratinocytes


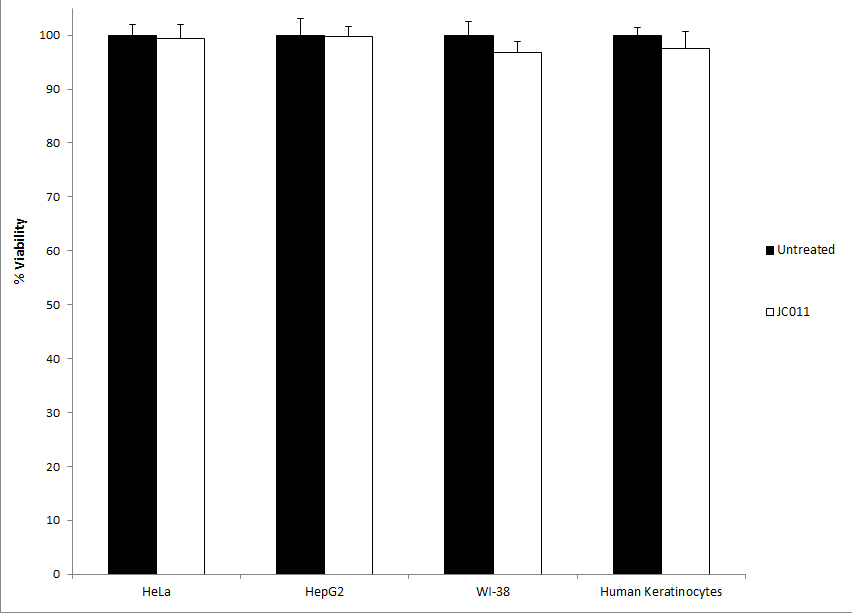


Figure 7 in File S1: Immunomarker FACS analysis of PSC cell surface markers


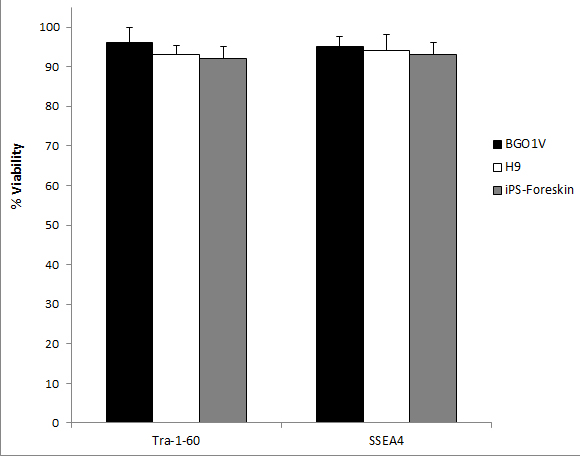


Figure 8 in File S1: DTT treatment does not induce ER stress cell death in BGO1V


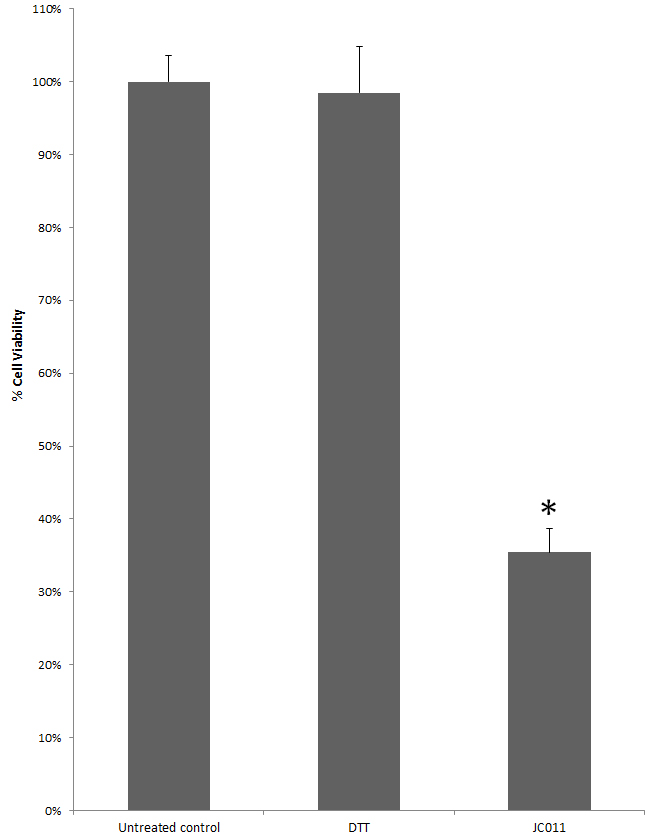


Figure 9 in File S1: Surrogate ROS levels in NCCIT following ATF4 and DDIT3 siRNA knockdown


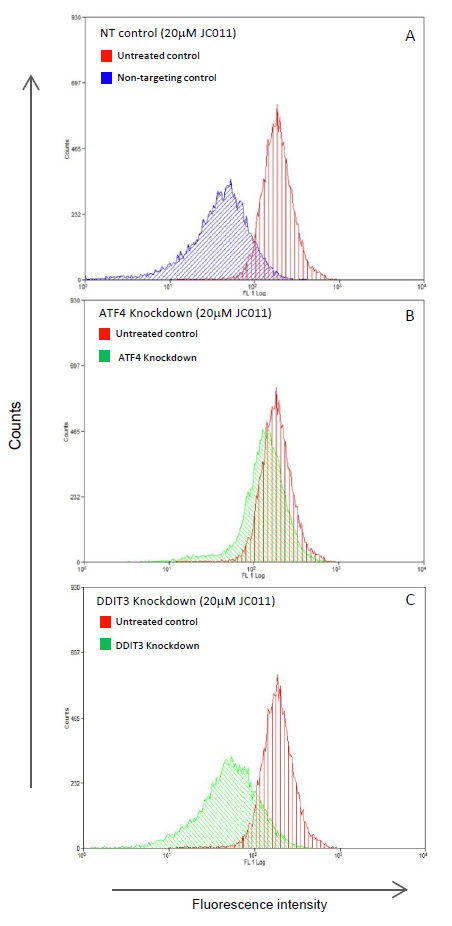


Figure S10 in File S1: Synthetic Procedure for Analogues JC005, JC011, JC040, JC048-050.

Figure S11 in File S1: Synthetic Procedure for Analogues JC007, JC010 and JC017.
